# Thermal Conductivity and Cure Kinetics of Epoxy-Boron Nitride Composites—A Review

**DOI:** 10.3390/ma13163634

**Published:** 2020-08-17

**Authors:** John M. Hutchinson, Sasan Moradi

**Affiliations:** Departament de Màquines i Motors Tèrmics, ESEIAAT, Universitat Politècnica de Catalunya, Carrer Colom 11, 08222 Terrassa, Spain; sasan.moradi@upc.edu

**Keywords:** epoxy, boron nitride, thermal conductivity, cure kinetics

## Abstract

Epoxy resin composites filled with thermally conductive but electrically insulating particles play an important role in the thermal management of modern electronic devices. Although many types of particles are used for this purpose, including oxides, carbides and nitrides, one of the most widely used fillers is boron nitride (BN). In this review we concentrate specifically on epoxy-BN composites for high thermal conductivity applications. First, the cure kinetics of epoxy composites in general, and of epoxy-BN composites in particular, are discussed separately in terms of the effects of the filler particles on cure parameters and the cured composite. Then, several fundamental aspects of epoxy-BN composites are discussed in terms of their effect on thermal conductivity. These aspects include the following: the filler content; the type of epoxy system used for the matrix; the morphology of the filler particles (platelets, agglomerates) and their size and concentration; the use of surface treatments of the filler particles or of coupling agents; and the composite preparation procedures, for example whether or not solvents are used for dispersion of the filler in the matrix. The dependence of thermal conductivity on filler content, obtained from over one hundred reports in the literature, is examined in detail, and an attempt is made to categorise the effects of the variables and to compare the results obtained by different procedures.

## 1. Introduction

**Figure 1:**
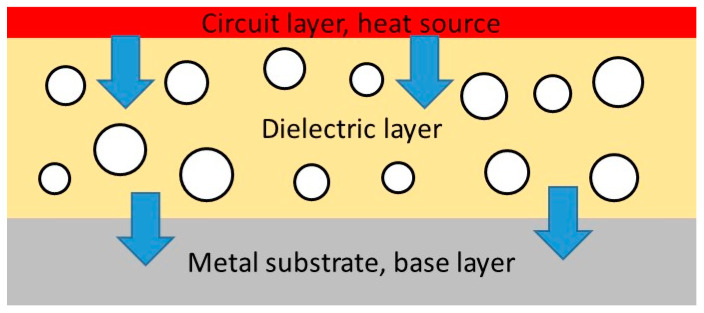
Schematic illustration of an insulated metal substrate (IMS).

Power electronics devices and other applications, such as solid-state relays and light emitting diodes (LED) in which high power density occurs, require an efficient means for the removal of the heat generated during operation. This has become increasingly important because the frequency of operation and power levels have risen in recent years. A good example of this is a consequence of the advances made in respect of LEDs, where technological developments have already brought three watt and five watt LEDs to the market-place, with even higher wattages predicted within the next few years [1]. Examples of applications involving ever increasing power levels include illuminated signs and displays; LED headlights and power steering in the automotive industry; and switches, relays, inverters and power supplies in the power electronics industry. The heat generated by these high power densities results in an increase in the temperature of the devices, which can have a detrimental effect on performance and significantly reduce the lifetime of operation. There are various rules of thumb to estimate the effect of such an increase in operating temperature, for example that the failure rate of an electronic device doubles with every 10 °C increase [2], or that every increase in operating temperature of 10% reduces the service life by 50% [3]. It is crucially important to be able to remove the heat from such devices, and considerable effort has been devoted to this end in recent years. In practice, this is commonly achieved by the use of insulated metal substrates (IMS), in which the copper conductor is bonded to a metal substrate by a dielectric layer, as shown schematically in Figure 1.

There are many commercial IMS systems available, from companies such as Bergquist [1], AI Technology [2], Technoboards Kronach [4] and Ventec International Group [5]. At the heart of all these systems is the dielectric layer, which must offer a variety of properties, including electrical insulation, thermal conductivity, ease of processing and adhesive strength. There are some additional requirements of this dielectric layer, for example the need for the thermal expansion coefficient to be matched to that of the other components, as well as limitations on its thickness, which is usually in the range from 50 to 150 μm. However, the most important aspect from the perspective of this review is thermal conductivity, while bearing in mind the need to accommodate the other requirements. Adhesion is generally achieved by using an epoxy resin as the matrix material, which satisfies the requirement of electrical insulation but has a very low thermal conductivity, typically around 0.2 W/mK [6]. In order to increase the thermal conductivity, a filler is commonly added. This filler must be thermally conductive but electrically insulating, and examples of suitable materials include oxides, such as Al_2_O_3_, SiO_2_ and ZnO [7,8,9,10]; carbides, such as silicon carbide (SiC) [11,12]; and nitrides, such as AlN, BN and Si_3_N_4_ [13,14]. In this way, commercial IMS products are available that offer thermal conductivities typically in the range from 0.8 to 4.2 W/mK [1,2,3,4,5], though values as high as 7.0 [5] and 7.5 W/mK can be found [1]. This represents a significant improvement in the range of from 0.2 to 0.4 W/mK for the traditional FR-4 printed circuit board technology.

**Figure 2:**
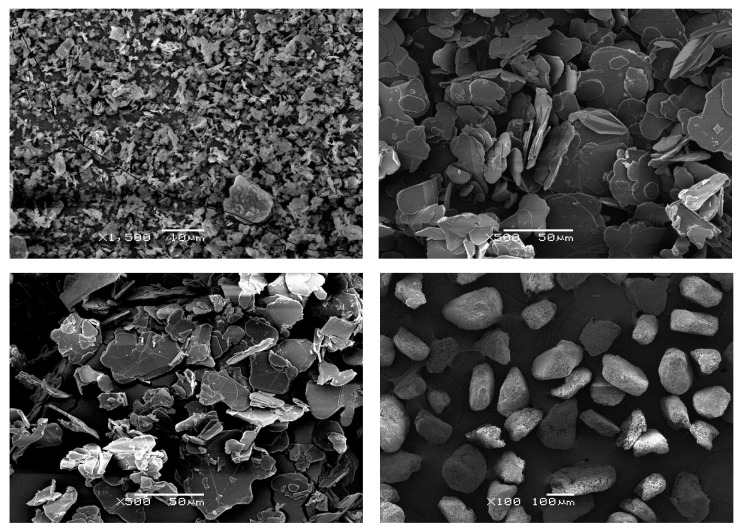
SEM micrographs of boron nitride (BN) particles from Saint-Gobain [17]: top left, 2 μm platelets, PCTP2; top right, 30 μm platelets, PCTP30; bottom left, 180 μm platelets, PCTP30D; bottom right, 120 μm low density agglomerates, PCTL7MHF.

There is clearly a wide variety of candidate materials for the dielectric layer, but in this review we consider only boron nitride (BN), on account of its wide application and that it has one of the highest values of thermal conductivity for electrically insulating materials. Hexagonal boron nitride is a white solid material, often referred to as “white graphite” because it has the same plate-like hexagonal structure as graphite and has lubricating properties but, in contrast to graphite, BN is a good electrical insulator and is therefore suitable for IMS application. The thermal conductivity of hexagonal BN is reported to be up to 30 W/mK in the direction perpendicular to the hexagonal planes, and up to 600 W/mK parallel to these planes [15,16]. BN is usually available in the form of powder, the powder particles being either in the form of platelets or agglomerates, and with a range of possible sizes. An example of the grades on offer can be seen in the product list provided by Saint-Gobain [17], which includes platelets with average particles sizes from 2 to 180 μm, and agglomerates, both low density and high density as well as spherical powders, with average sizes from 80 to 300 μm. Figure 2 shows scanning electron microscopy (SEM) micrographs of some of these grades of BN particles.

The fabrication procedure for epoxy-BN composites involves mixing the epoxy resin, curing agent and BN particles, as well as some other components such as the catalyst or accelerator in some cases, and then effecting the cure, usually isothermally or in a series of isothermal stages. Although the thermal conductivity of the final cured product is essentially determined by the BN particles, it might be expected that the curing process could affect the interaction between the epoxy matrix and the BN filler particles. In particular, the matrix-filler interface plays a crucial role in the development of the thermal conductivity in the composite, and any interaction between the matrix and filler would be evident in the curing reaction. Thus, we begin with a review of the cure kinetics of epoxy composites in general, and then of epoxy-BN composites in particular. We then examine in more detail how the thermal conductivity of the cured composites depends on the numerous parameters involved in their fabrication.

## 2. Cure Kinetics

### 2.1. General Aspects

The fabrication of epoxy-BN composites requires the curing of the epoxy matrix. In practice, this will usually be an isothermal cure, either in a single stage or in two stages, with an initial partial cure at a temperature less than the glass transition temperature of the fully cured system, *T*_g∞_, followed by a post-cure at a temperature higher than *T*_g∞_. It might be expected that the details of the cure procedure, for example the isothermal cure temperature, could have an influence on the properties of the cured composite, such as the mechanical and thermal properties, and including the thermal conductivity. For this reason, it is interesting to investigate the cure kinetics, the analysis of which usually involves not only isothermal but also non-isothermal cure.

The experimental study of cure kinetics is typically made by differential scanning calorimetry (DSC), and the analysis is based on one of a number of relatively simple kinetic equations. All of these equations describe the time (*t*) dependence of the degree of cure (α), the latter being determined experimentally as the ratio of the heat of reaction at time *t*, Δ*H*(*t*), to the total heat of reaction, Δ*H*_∞_, for a fully cured sample:

(1) $$\alpha =\frac{\Delta H\left(t\right)}{\Delta H_{\infty }}$$

The heat of reaction at time *t* is obtained from the area under the DSC curve of heat flow up to this time, while the total heat of reaction requires the area under the complete cure curve, and both are usually expressed per unit of mass of sample, in J/g. If no vitrification occurs during the curing process, then the cure can be described by a chemical rate equation in which the rate of cure is both temperature (*T*) dependent and a function of the degree of cure:

(2) $$\frac{d\alpha }{dt}=k\left(T\right)f\left(\alpha \right)$$

In this equation, *k*(*T*) is the rate constant and depends on temperature according to an Arrhenius equation:

(3) $$k\left(T\right)=A\exp\left(-\frac{E}{RT}\right)$$

where *A* is the pre-exponential factor or frequency factor, *R* is the universal gas constant and *E* is the activation energy.

The function *f*(α) depends on the kinetic model considered most appropriate for the given circumstances. For epoxy cure, the autocatalytic model is one of the most widely used, and the function can be expressed by the Sestak–Berggren equation:

(4) $$f\left(\alpha \right)=\alpha^{m}{\left(1-\alpha \right)}^{n}$$

where the kinetic exponents *m* and *n* often sum to approximately two. One problem with this equation is that, without an initial non-zero value for the degree of cure, α, the reaction will never start. Furthermore, the equation requires that the initial reaction rate be zero, whereas this is not necessarily the case. An alternative equation for the chemical reaction rate, which overcomes these problems, is the Kamal equation:

(5) $$\frac{d\alpha }{dt}=\left(k_{1}+k_{2}\alpha^{m}\right){\left(1-\alpha \right)}^{n}$$

where both rate constants *k*_1_ and *k*_2_ have Arrhenius temperature dependences, with different activation energies and pre-exponential factors. The initial reaction rate in the Kamal equation is given by *k*_1_.

The above equations have been rather widely used for the analysis of the cure kinetics of unfilled epoxy systems, with a variety of different resins, curing agents and initiators or accelerators. This approach has been adopted by many authors (e.g., [18,19,20,21,22,23,24]) to interpret aspects of the reaction and of the cured system in terms of the parameters of the model (*A*, *E*, *m* and *n*).

### 2.2. Epoxy Composites

In epoxy composite systems, on the other hand, there is the additional effect of the filler to be considered, and here two different aspects can be identified: the effect of the filler on the cure kinetics and the effect on the properties of the cured composite. Considering the latter aspect, the purpose of the filler is clearly to modify the properties of the composite. For example, glass, carbon or aramid fibres are incorporated into an epoxy matrix in order to improve the mechanical properties, and many fillers are added to reduce the thermal expansion coefficient or even simply to reduce the cost of the material. In this context, the boron nitride filler is added in order to enhance the thermal conductivity of the composite. Besides these changes deliberately induced in the properties of the overall composite material, there is the question of the effect of the filler on the epoxy matrix, and here the situation is not well understood. For example, it is commonly believed that increasing the filler content will lead to an increase in the glass transition temperature, *T*_g_, of the cured composite because the filler particles will provide a restriction of the molecular mobility of the epoxy matrix. However, a literature search using the key words or phrases “effect of fillers”, “glass transition temperature” and “epoxy” gives nearly 400 results in the Web of Science database, many of which conclude that there is no significant effect of filler on *T*_g_, though there are reports of both an increase and a decrease in *T*_g_ on the addition of a filler, as the following examples illustrate.

Many years ago, Filyanov [25] alluded to the generally held view that fillers restrict molecular mobility when he wrote “in contradistinction to the majority of cases where the glass transition temperature of an epoxy resin rises during filling”, referring to his own results for an epoxy resin filled with glass microspheres, which instead featured a marked reduction in *T*_g_. This effect was attributed to an “adsorptive-adhesional interaction between resin components and the filler surface”. The dominant role of the matrix-filler interface, and in particular “enhanced polymer dynamics”, was also cited by Sun et al. [26] to explain why they observed a decrease in *T*_g_ for epoxy-silica nanocomposites in comparison with the corresponding composites with micron-sized fillers. However, it is not clear why these very general interface attributes should apply in these situations and not in others. Indeed, Stevens and Richardson [27] investigated a silica-filled epoxy-anhydride system, for which silanol groups at the silica surfaces were potential hydrogen-bonding sites for hydroxyl and epoxide groups, and therefore with much greater potential for filler-matrix interaction, but they concluded that there was no significant filler effect on *T*_g_ in the fully cured composites. That the situation is complicated by other interfacial aspects was highlighted more recently in a review [28] of the effect of carbon nanotubes on the *T*_g_ of epoxy nanocomposites; the bundling tendency of single-wall nanotubes can lead to a reduction in *T*_g_, whereas *T*_g_ increases or remains constant for multi-wall nanotubes.

On the other hand, the following examples are representative of many (probably the majority of) reports which suggest that *T*_g_ is essentially independent of the filler content in epoxy composites. Linec and Music [29] investigated the effects of the addition of silica of different shape, specific surface area and chemical structure, including crystalline and fused silica, in epoxy moulding compounds and found that *T*_g_ remained almost constant. Dorigato and Pegoretti [30] introduced both carbon black (CB) and carbon nanofibers (NF) into an epoxy matrix with in situ generated silver nanoparticles and concluded that *T*_g_ was “slightly reduced”; for example, for 4 wt.% nanofiller the reduction was about 5.5 °C for the CB nanofiller, but only about 2.6 °C for NF. In particular, these authors could not detect any clear trend of *T*_g_ with the relative proportions of the two carbonaceous nanofillers, in agreement with their earlier observations with various other nanofilled thermosets. They attributed this result to two competing effects of the nanofillers: an increase in *T*_g_ due to “chain blocking mechanisms”, and a reduction in *T*_g_ due to a reduction in the degree of cross-linking as a consequence of matrix-filler interactions and increased viscosity.

The complexity of the situation is illustrated by another result. In ternary epoxy nanocomposites filled with 5 wt.% titanium dioxide nanoparticles and halloysite nanotubes (HNT), Vijayan et al. [31] found a small decrease (about 3 °C) in *T*_g_ for epoxy-TiO_2_ composites, a significant increase (>10 °C) for epoxy-HNT composites, but essentially no change for the ternary composites. While the authors asserted that HNT can restrict the segmental motion of cross-links near the matrix-HNT interface, thus giving rise to the observed increase in *T*_g_, they did not explain why *T*_g_ remained essentially constant for the ternary composites. More generally, Kang et al. [32] concluded that composites with a weak filler-matrix interface show essentially no change in *T*_g_, whereas a strong interface promotes an increase in *T*_g_.

In the above discussion of the properties of the epoxy matrix in cured composites, the glass transition is associated with the network structure of the cured epoxy, which is developed during the course of the previous curing schedule. Consequently, we turn now to the second aspect mentioned above, namely the effect of the filler type and content on the cure kinetics in epoxy composites. First, we will consider the situation in general, before reviewing the curing process of epoxy-BN composites in particular.

Although many of the fillers used for epoxy composites are essentially inert with respect to epoxy, and hence might not be expected to have any effect on the cure reaction, in practice there are several chemical species which can occur at the surface of filler particles that can influence the reaction. Consequently, it is common to see reports on the effect of fillers in an epoxy composite cure, and the most obvious effect is whether the reaction is accelerated or retarded by the presence of the filler. This can be assessed simply by examining whether there is a reduction or increase, respectively, in the peak temperature, *T*_p_, during a non-isothermal cure or in the time to the peak exotherm, *t*_p_, in an isothermal cure. Alternatively, the kinetic Equations (1)–(5) can be used to fit the cure curves and obtain the values of the parameters, in particular the activation energy, *E*, and the pre-exponential factor, *A*. In this respect it should be pointed out that it is sometimes assumed that a reduction in *E* implies an acceleration of the reaction, but this is incorrect: the reaction rate is described by the rate constant *k*, which is a function of both *E* and *A*. Thus, it is possible for there to be a reduction in *E* and yet a slowing down of the reaction as a consequence of a concurrent reduction in *A*. Comment on this will be made when discussing some of the examples considered below.

The most common observation is that the cure reaction of epoxy composites is accelerated by the addition of fillers (e.g., [33,34,35,36,37,38,39,40,41,42,43]), most of these systems being based on DGEBA epoxy resin, and cured with methylene dianiline [33,34], anhydride [36,42], or amines [35,39,43]. A wide variety of fillers is included here: zeolite [33,34,36], zirconium tungstate [37], alumina [39,42], barium ferrite/aniline [41], carbon black and carbon fibres [35], aluminium nitride [40], silica [38] and mallow fibres [43]. The advance of the curing process is typically identified by a reduction in the peak exotherm temperature in a non-isothermal cure or by a decrease in the time to the peak exotherm in an isothermal cure. However, while a systematic effect of filler content might be expected, in some cases [37,40,42] the effect reported is an initial acceleration, apparently simply as a consequence of the addition of the filler, but with no further significant or systematic change as the filler content is further increased.

Nevertheless, the acceleration effect of adding fillers is not always clearly evident. For example, Olivier et al. [44] monitored the cure kinetics of a polyepoxy cured with polyamineaniline and filled with either polyamide-12 or a ceramic. During non-isothermal cure, the peak temperature clearly decreased as the content of either filler increased, indicating an acceleration effect. However, when these authors analysed the isothermal cure, they found that, for a given cure time, the degree of cure was lower for the filled samples than for the pure epoxy, and hence concluded that the fillers slow down the reaction. However, there is some inconsistency, in that the heat of reaction, apparently per unit mass of epoxy, decreased as the filler content increased, which could affect the isothermal cure analysis. Likewise, Sanctuary et al. [45] reported different effects in non-isothermal and isothermal cures for a DGEBA epoxy resin filled with alumina nanoparticles and cured with a triamine. Since the isothermal cure was carried out at 25 °C, which is less than *T*_g∞_, it involves vitrification, which is detected by temperature-modulated DSC. With increasing filler content in the isothermal cure, the magnitude of the heat flow increased and the vitrification time was reduced, indicating an acceleration of the reaction by the filler. On the other hand, these authors obtained the surprising result that the non-isothermal cure kinetics is essentially unaffected by filler content and discuss this in terms of an interphase region between the nanoparticles and the epoxy matrix, in which there is reduced molecular mobility.

Differences between the effects of fillers in non-isothermal and isothermal cure can also be inferred from the early results of Dutta and Ryan [46]. The isothermal cure of a DGEBA epoxy resin cured with a diamine and filled with carbon black or silica at 6 wt.% content was advanced relative to the unfilled system, and more so for the former filler. However, in the non-isothermal cure, while for the same 6 wt.% content the peak exotherm temperature was reduced for both fillers compared to the unfilled system, and more so for the carbon black, there was no systematic effect of other filler contents. For carbon black the cure was apparently retarded for 1 wt.% and 4 wt.% but was slightly advanced for 2 wt.%, while for silica it was also retarded for 4 wt.% content. In the non-isothermal cure of a DGEBA epoxy filled with quartz flour, de Miranda et al. [47] also reported that the effect of the filler on cure kinetics depends on the range of the filler content. For up to 10 wt.% filler, the rate constants for the filled and unfilled systems are the same for any given degree of cure, whereas at 20 wt.% and 30 wt.% content, the rate constants are approximately equal but lower than that for the unfilled system. This suggests that a filler content greater than 10 wt.% will slow down the reaction, in contrast to the generally observed effect. However, the reported peak exotherm temperatures at 10 °C/min for the filled systems, from 2 to 30 wt.%, did not vary systematically, and their variation is much smaller than the difference between their average value and that of the unfilled resin. Accordingly, it is difficult to draw any firm conclusions from these results.

There are a few other reports of a decrease in the cure rate as a consequence of the presence of fillers. Omrani et al. [48] studied a DGEBA epoxy filled with barium carbonate at up to 15 parts per hundred resin (phr) and found that the peak exotherm temperature increased by 10 °C for the highest filler content, indicating a slowing down of the reaction rate. However, a large variability in the heat of reaction between repeated experiments suggested a heterogeneous filler dispersion, which could have influenced the cure kinetics and may also have been responsible for an anomalous variation of the glass transition temperature. Zabihi et al. [49] investigated a DGEBA epoxy, anhydride cured and filled with polythiophene nanoparticles, and found that in non-isothermal cure the peak exotherm temperature increased by between 10 and 15 °C with an addition of only 1% nanoparticles. Just as for Omrani et al. [48], however, they also reported a large variation in the heat of reaction for filler loadings greater than 1%, again attributed to an inhomogeneous distribution and agglomeration of the nanoparticles. Similar results were presented by Karasinski et al. [50], who studied the cure kinetics of a DGEBA epoxy cured with *o*-tolyl biguanidine and filled with nanoparticles of either γ-alumina or zinc oxide at a loading of 3 phr, corresponding to volume percentages of 1.2 and 0.6, respectively. These authors also reported a slight tendency of the nanoparticles to cluster, with the more homogeneous distribution being found for zinc oxide. For these epoxy composites, the peak exotherm temperature increases markedly, for example by more than 10 °C for the alumina composites and by more than 30 °C for the zinc oxide composites, at the heating rate of 10 K/min. However, this is accompanied by a significant increase in the heat of reaction for the nanocomposites, which is attributed to a higher cross-linking density. This is consistent with an increase in *T*_g∞_ for the nanocomposites with respect to the unfilled system, but probably indicates that the presence of benzoin as a degassing agent and of poly(*n*-butylacrylate) as a plasticizer have modified the cure reaction, thus making it difficult to distinguish the effect of the filler nanoparticles on the cure kinetics.

In summary, it is probably true to say that it is not possible to draw any firm conclusions from the reports in the literature where the cure of epoxy composites is apparently retarded by the addition of the filler particles: the heat of reaction is often not constant, there are different effects in isothermal and non-isothermal cure, there are unsystematic variations with the filler content, and sometimes inhomogeneous dispersion or agglomeration of filler particles occurs.

There are also reports of the fillers having no effect on the cure kinetics of epoxy composites. Tarrío-Saavedra et al. [51] show DSC non-isothermal curves for an amine-cured epoxy filled with up to 50 wt.% fumed silica, where the peak exotherm temperature is unaffected by the filler content. As the title of their paper suggests, however, there are some unusual observations, which throw some doubt on the interpretation of the effect of the filler on the cure kinetics. For example, the heat of reaction varies in a non-systematic way with filler content, which the authors themselves interpret as agglomeration of the nanoparticles and a possible loss of stoichiometry in the reaction. On the other hand, Ghaffari et al. [52] investigated the cure kinetics of a DGEBA epoxy cured with polyaminoamide, but with only a single and very small (1 wt.%) content of either micro- or nano-sized zinc oxide particles. Furthermore, they used only isothermal cure, for which there is very little difference between the iso-conversional cure times as a function of the inverse temperature for the different composite systems. These plots, as well as a model-fitting procedure, allowed the evaluation of the activation energy, *E*, which decreased for the micro-composite and, to an even greater extent, for the nano-composite. This reduction in *E* should not, however, be associated with an advance of the reaction for the filled systems, as it was accompanied by a significant reduction in the pre-exponential factor, *A*, and again to a greater extent for the nano-filler compared with the micro-filler.

Finally, another effect, namely that of the surface treatment of the filler particles, was investigated by Harsch et al. [53], who analysed the cure kinetics of a cycloaliphatic epoxy cured with anhydride and filled with a variety of fillers, including silica, both with and without a surface treatment. These authors found that the addition of the surface-treated filler reduced the activation energy, *E*, and the pre-exponential factor, *A*, while they remained unaffected by the untreated filler, and they concluded that the surface-treated filler accelerates the reaction. These authors did not, however, offer any explanation for this observation.

**Figure 3:**
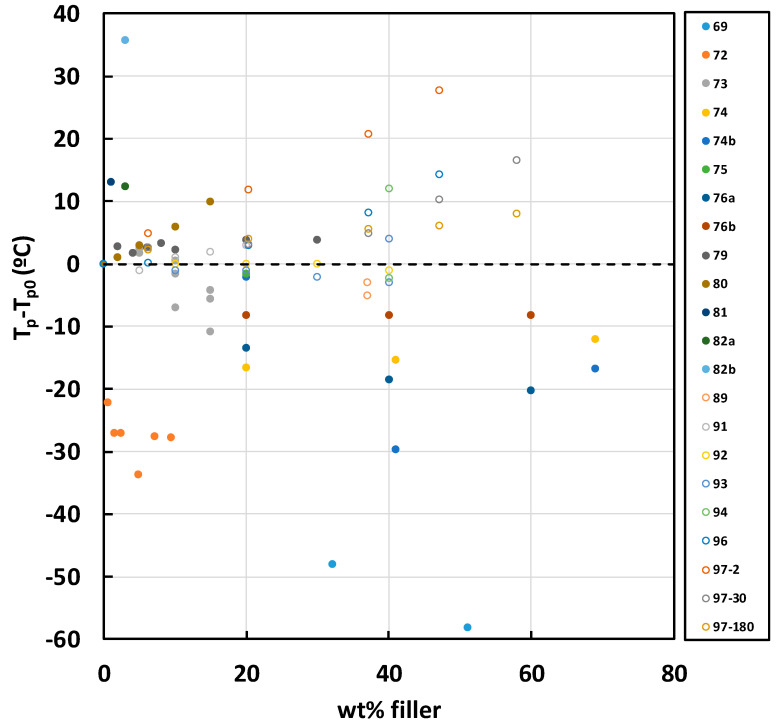
Effect of filler content in epoxy composites on the peak temperature, in non-isothermal cure at 10 K/min, relative to that of the unfilled system. Heating rate 10 K/min. Open symbols: boron nitride filler; filled symbols: other fillers.

**Figure 4:**
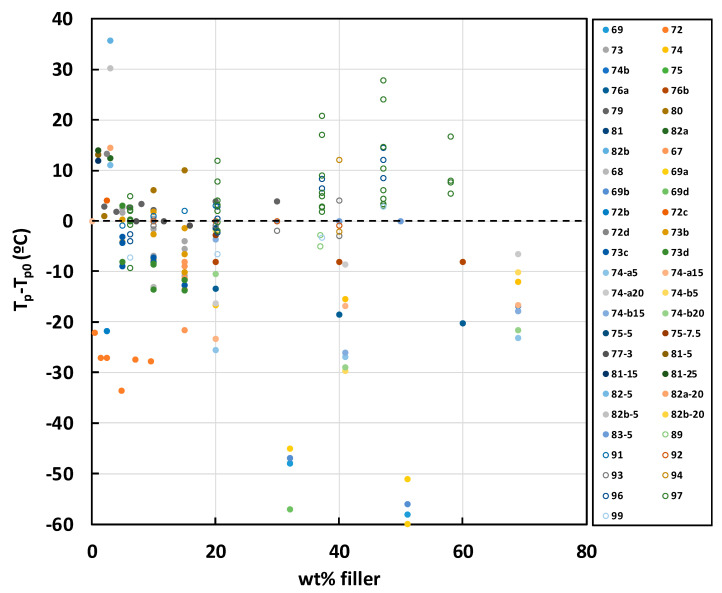
Effect of filler content in epoxy composites on the peak temperature in non-isothermal cure, relative to that of the unfilled system. No distinction is made here between results obtained at different heating rates. Open symbols: boron nitride filler; filled symbols: other fillers.

A compilation of all the results for non-isothermal cure is given in Figure 3 and Figure 4. The filled symbols correspond to the data obtained for epoxy composites with fillers other than BN, while the open symbols correspond to epoxy-BN composites. In Figure 3, data are included for the heating rate of 10 K/min only, whereas in Figure 4 all heating rates are included. The correspondence between the symbols used in Figure 3 and Figure 4 and the references from which the data were obtained is given in the legends, but can be seen more clearly by accessing Supplementary Material 1. For filler particles other than BN, the filled symbols in Figure 3 and Figure 4 demonstrate the tendency for the peak temperature *T*_p_ to decrease with the addition of filler particles in the epoxy composites, particularly at higher filler contents.

In the light of the compilation and discussion above, where it might be concluded that the cure reaction of epoxy composites is usually accelerated by the addition of fillers, it is interesting to consider the explanations that are given for why this acceleration might occur. In nearly all cases in which acceleration of the reaction occurs, this is attributed to the catalytic effect of hydroxyl groups present on the surface of the various filler particles, be they zeolite [33,34,36], zirconium tungstate [37], nano-SiO_2_ [38], nano-alumina [39], or nano-AlN particles [40]. It should be noted that there is no need for the surface of the filler particles to be modified for there to be a catalytic effect; for example, for the epoxy-zeolite composites [33,34,36], the filler was natural zeolite without any surface treatment. Likewise, Sanctuary et al. [45] attributed the acceleration of the isothermal cure of epoxy/nano-alumina composites to the surface catalytic action of –OH groups and argued that the lack of any acceleration effect in non-isothermal cure was a consequence of the reduction of the molecular mobility by the “interphases”. More generally, Dutta and Ryan [46] considered that the acceleration of epoxy cure when filled with carbon black was due to the presence of numerous constituents found on the surface of the carbon black, principally phenolics, carboxylics, quinones, hydroquinones and lactones. In contrast, they attributed the lack of any catalytic effect when the epoxy is filled with silica to either the lower specific surface area (SSA) or the relatively complex-free surface of the silica.

In some cases, the catalytic effect is reported to be modified by surface treatment of the filler particles, though there is no consensus on the effect. Haman et al. [37] consider that silane functionalisation of zirconium tungstate particles retards the cure as a consequence of the shielding of –OH groups by silane, while Yu et al. [40] consider that the catalytic effect of their silane treated nano-AlN particles results from both –OH and –NH_2_ groups on the surface. The catalytic effect of the amino group was also proposed by Bi et al. [42] for the cure of their epoxy composites filled with amino-functionalised alumina particles. Similarly, Harsch et al. [53], as noted above, only found an acceleration of the cure rate when the filler particles are surface treated.

Some other explanations have been given for the acceleration of the cure for some particular circumstances. Wu et al. [35] observed that the incorporation of activated carbon fibres into their composites accelerated the reaction, and that the activation of the carbon fibres simultaneously increased their SSA significantly. On the other hand, when the carbon fibres were ozone treated, there was neither an acceleration of the cure nor any significant change in the SSA of the fibres. Accordingly, these authors associated the acceleration of the reaction with an increase in the SSA, an observation that accords with that of Dutta and Ryan [46]. In another particular situation, for epoxy/barium ferrite/polyaniline composites, Saad et al. [41] considered that the protonated amine-imine groups of polyaniline act as epoxy-opening initiators to accelerate the cure reaction. Finally, in a quite different epoxy composite system, in which mallow fibres give rise to a very slight acceleration of the cure, Nascimento et al. [43] concluded that mallow fibres act as “nucleation sites”.

### 2.3. Epoxy-BN Composites

The general aspects of the cure kinetics of epoxy composites discussed above can now be compared with the particular behaviour of epoxy-BN composites. However, apart from the contributions of two particular research groups—Isarn et al. and Hutchinson and co-workers—considered in more detail below, there is rather little work reported on the cure kinetics of epoxy-BN composites, despite the fact that such composites are very widely studied in respect of their thermal conductivity. Furthermore, in some instances [54,55,56], unfortunately, the calorimetric cure data are either poorly presented or confusing, to the extent that no reliable conclusions can be drawn about the influence of the filler on the cure kinetics. The results of Teng et al. [57] are more interesting. These authors used a DGEBA epoxy matrix cured with diamino diphenyl sulphone and filled with combinations of BN, both unmodified and modified with a zirconate coupling agent, and both functionalised and non-functionalised multiwall carbon nanotubes (MWCNT). The peak temperature in non-isothermal cure at 5 °C/min decreased slightly, by about 3 °C and 5 °C for the modified and unmodified BN, respectively, for a loading of 25 vol% BN, which is consistent with the usually observed acceleration of the cure reaction with added filler, as discussed above. However, at the same time there was a large difference between the heats of reaction for the composites with unmodified fillers and those with the modified and functionalised fillers, the heat of reaction for the latter being about twice that of the former. These authors allude to the steric hindrance of the filler particles as an explanation for these observations, although this might be expected to slow down rather than accelerate the reaction, and also conclude that the reactive sites of the functionalised hybrid filler react with the epoxy resin, which does not occur for the pristine hybrid filler. This additional reaction is believed to reduce the free volume and hence improve the matrix-filler interface, which was correlated with an increase in thermal conductivity. This interpretation is interesting and is a good illustration of how calorimetric cure data can be related to the thermal conductivity of the cured composite; further examples of such a relationship will be presented below. Nevertheless, the thermal conductivities achieved by these authors remain rather low for the BN filler loadings used, as will be seen in Section 3.1 of this review. Another report, by Wu et al. [58] on bisphenol-A epoxy composites filled with nano-BN particles, is ambiguous and inconsistent in reporting the dependence of the peak exotherm temperature in non-isothermal cure on BN content, so no conclusions can be drawn from this work.

For various reasons, it is not possible to draw any reliable conclusions from the references discussed above [54,55,56,57,58] about the effect of the addition of BN particles on the cure kinetics of epoxy-BN composites, for comparison with the generally observed acceleration. It is, therefore, interesting to note that Isarn et al. [59,60,61,62] and Hutchinson and co-workers [63,64,65,66,67] present many results which show the opposite effect, namely a retardation of the cure upon addition of BN, and accordingly these are examined now in some detail. There are some differences in the matrix materials and fillers used by the two groups. The former group used two types of epoxy: DGEBA and a cycloaliphatic resin, 3,4-epoxy cyclohexylmethyl 3,4-epoxycyclohexane carboxylate (ECC), and effect the cure reaction in two separate ways. In most of their work, these authors induced epoxy homopolymerisation by cationic initiation, but in one of their studies they used a thiol, pentaerythritol tetrakis (3-mercaptopropionate) (PETMP), as a cross-linking agent, with 4-(N,N-dimethylamino)pyridine (DMAP) as the initiator. The filler is principally BN, but carbon nanotubes (CNT) were added occasionally.

For composites with a DGEBA epoxy matrix, for which only an antimonate cationic initiator was used, BN particles of around 6 μm size in non-isothermal cure gave rise to a very slight increase in the peak exotherm temperature, *T*_p_, from 120 °C to 123 °C, when increasing the BN content up to 20 wt.%; this was attributed to “the hindrance produced by the BN particles” or “the increased viscosity of the formulation” [59]. For a higher BN content of 40 wt.%, with the same epoxy/initiator system, a much greater increase in *T*_p_, from 121 °C to 133 °C, was reported, essentially independent of whether or not 1 wt.% of CNT was also included in the composite [62]. This retardation of the cure upon addition of BN particles was attributed to “the dilution effect of the high quantity of filler”. Isoconversional analysis shows that the cure curves all follow a second order kinetic model, the retardation on the addition of BN particles being demonstrated by a reduction in the rate constant, *k*. Furthermore, the heat of reaction, measured in kJ/ee, and the glass transition temperature of the fully cured composite, *T*_g∞_, were both essentially independent of the filler content, indicating that the network structure was not influenced by the presence of the filler.

For the other epoxy matrix material, ECC, the peak exotherm temperature remains very much independent of the BN content. When the cure is cationically initiated [60,62], *T*_p_ either remains constant [60] or at most decreases by only 2 °C [62] for the non-isothermal cure of composites with 40 wt.% BN, though for the hybrid with 1 wt.% CNT a slightly greater decrease of 3 °C was observed. This was interpreted as indicating a cooperative effect between the two different fillers; possibly the protons formed during the oxidation of the CNT help to catalyse the ring-opening polymerisation. Likewise, when the ECC epoxy was cured with a thiol, PETMP, and initiated with DMAP, there was only a small change in *T*_p_ on addition of 40 wt.% BN, though the direction of the change depended on the BN particle size: for 6 μm particles there was a 3 °C decrease, whereas for 80 μm particles there was a 4 °C increase [61]. The authors concluded that DGEBA resin systems are likely to interact better with BN than ECC resin systems.

The dependence of the peak exotherm temperatures in non-isothermal cure for epoxy-BN composites is plotted together with the data for the other fillers in Figure 3 and Figure 4 as the open symbols. Here, it can be seen that the tendency is quite the opposite of that for the other fillers, though the reason for this is not clear.

In nearly all cases, Isarn et al. found that the heat of reaction per epoxy equivalent in the non-isothermal cure and the glass transition temperature of the fully cured systems, when it could be measured, were essentially independent of the filler content. They concluded that BN has an inert character with respect to the final network structure for both cationic epoxy homopolymerisation and when cured with a thiol. A slight decrease, from 120 kJ/ee for the unfilled epoxy to 114 kJ/ee for 40 wt.% of the 6 μm BN filler, and to 116 kJ/ee for the 80 μm BN filler in the ECC/PETMP system, was attributed to topological restrictions. The same explanation was given for the rather stranger decrease for cationic homopolymerisation, from 88 kJ/ee for the unfilled system to, initially, 69 kJ/ee for low BN content, but it then remained constant as the BN content increased.

**Figure 5:**
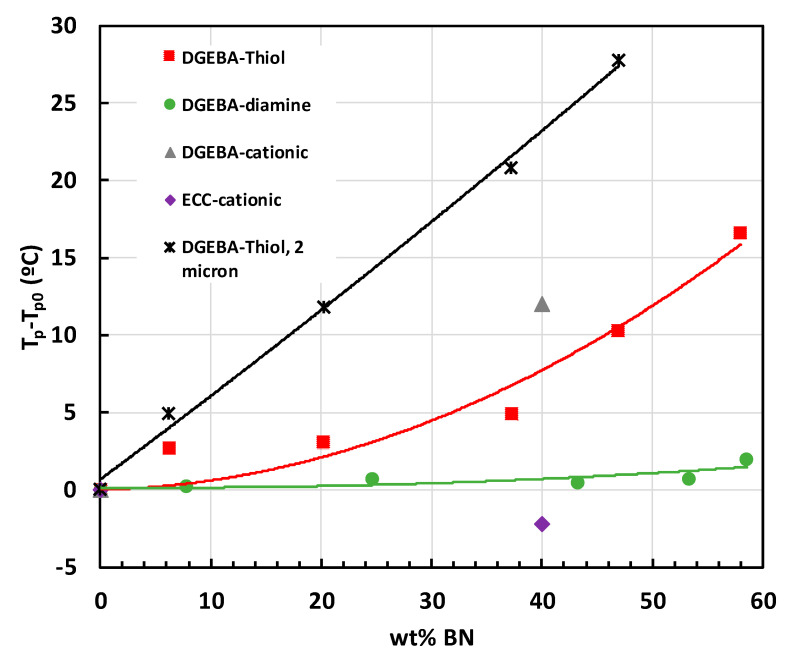
Dependence of peak temperature, *T*_p_, on filler content relative to unfilled epoxy, *T*_p0_, for epoxy-BN composites in non-isothermal cure at 10 K/min. 30 μm platelets: red squares, DGEBA-thiol [65]; grey triangle, DGEBA-cationic [62]; purple diamond, ECC-cationic [62]; green circles, DGEBA-diamine [65]. 2 μm platelets: black asterisks, DGEBA-thiol [65].

The conclusion that BN is an inert filler in epoxy composites was also reached by Hutchinson and co-workers in all their work on DGEBA epoxy composites cured with thiol (PETMP), where the heat of reaction and *T*_g∞_ were consistently independent of the BN content [63,64,65,66,67]. However, these authors also consistently observed an underlying retardation of the epoxy-thiol cure kinetics with the addition of BN particles, and they carried out a systematic study of the effects of various parameters in addition to the fundamental one of filler content, namely particle size [64,65], particle shape (i.e., platelets or agglomerates) [67], and surface modification [66]. Furthermore, they investigated the effect of using a diamine rather than thiol as the curing agent [63,64,65]. An illustration of some of these effects, and a comparison with the results of Isarn et al. [62] is shown in Figure 5.

The interesting aspects of this dependence of the cure kinetics on BN content are as follows. First, the DGEBA epoxy composite system cured either with thiol or cationically shows a similar retardation with the addition of 30 μm BN platelets. In contrast, the composite system based upon ECC epoxy resin does not display this retardation. Second, the DGEBA epoxy composite cured with diamine did not display any retardation of the cure with the addition of filler. This difference between the epoxy-thiol and epoxy-diamine composites was attributed to a Lewis acid-base interaction between the BN particles and thiol in the former, which does not exist in the latter [63,64,65]. The authors suggest that this could have a positive impact on the thermal conductivity of these composites as a consequence of the enhanced filler-matrix interaction. Third, there was a significant increase in the retardation effect when 2 μm BN platelets were used as the filler. Kinetic analysis of the cure curves for these 2 μm composites shows that the activation energy *E* decreased, which would be inconsistent with the observed retardation unless the pre-exponential factor *A* also decreased, and markedly so. Hutchinson and co-workers propose that the Lewis acid-base interaction between filler and matrix, coupled with the very large surface area of these much smaller particles, results in a large proportion of the epoxy matrix being immobilised at the particle surface [67].

**Figure 6:**
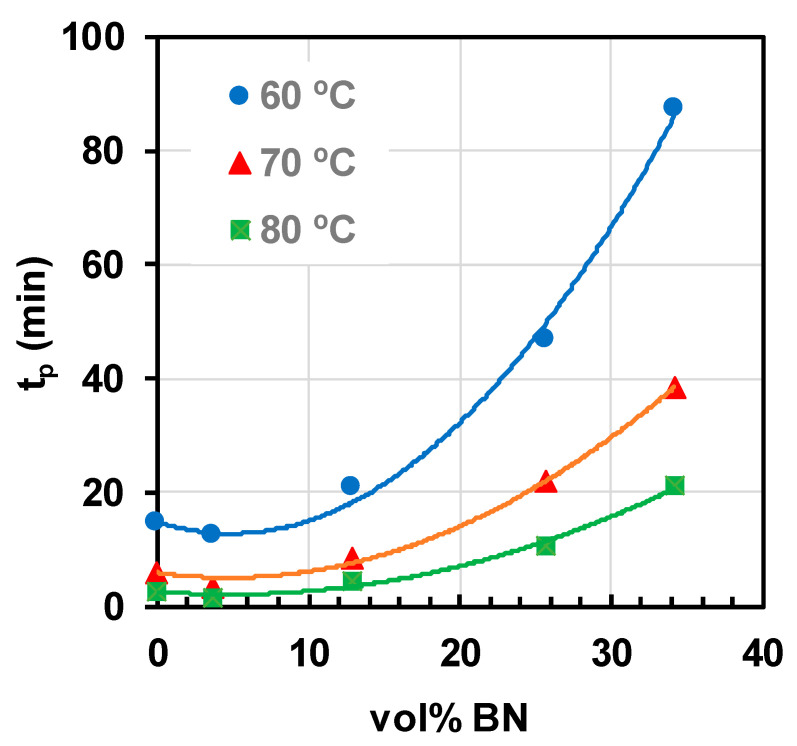
Dependence of time to peak exotherm, *t*_p_, on content of 2 μm BN platelets for epoxy-BN composites in isothermal cure at 60 °C (blue circles), 70 °C (red triangles) and 80 °C (green squares).

Besides this overall tendency for the cure reaction to be retarded by the BN filler reported by Hutchinson and co-workers, they also point out that, with a low BN content, there can be an acceleration effect. This is illustrated in Figure 6 for an epoxy-thiol system filled with 2 μm platelets and cured isothermally at three different temperatures [65], and is even more apparent in some other epoxy-BN systems [63,64,67]. In fact, this effect can also be seen in the cationic cure of DGEBA epoxy at the lowest BN concentration in the work of Isarn et al. [59]. It is argued that this could be a consequence of the improved heat transfer of the sample, hence representing a physical rather than a chemical effect.

## 3. Thermal Conductivity

### 3.1. Effect of BN Content

**Figure 7:**
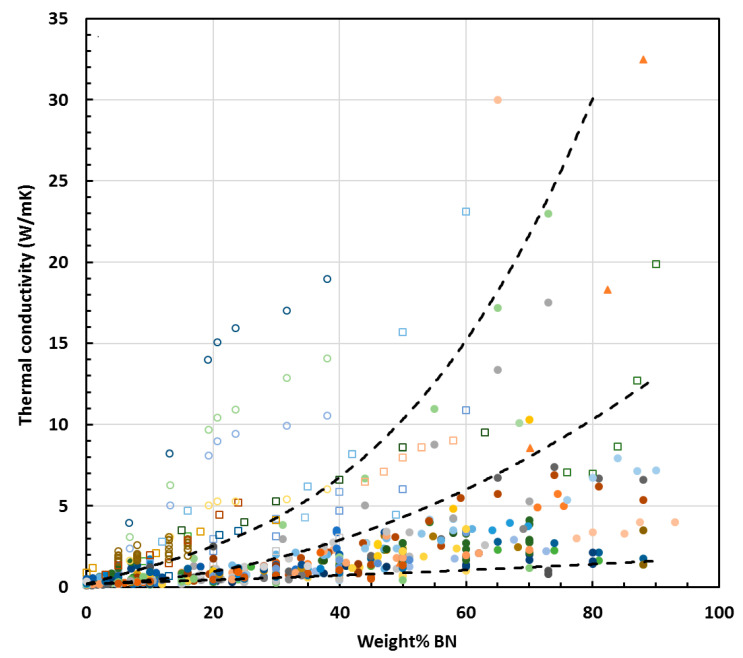

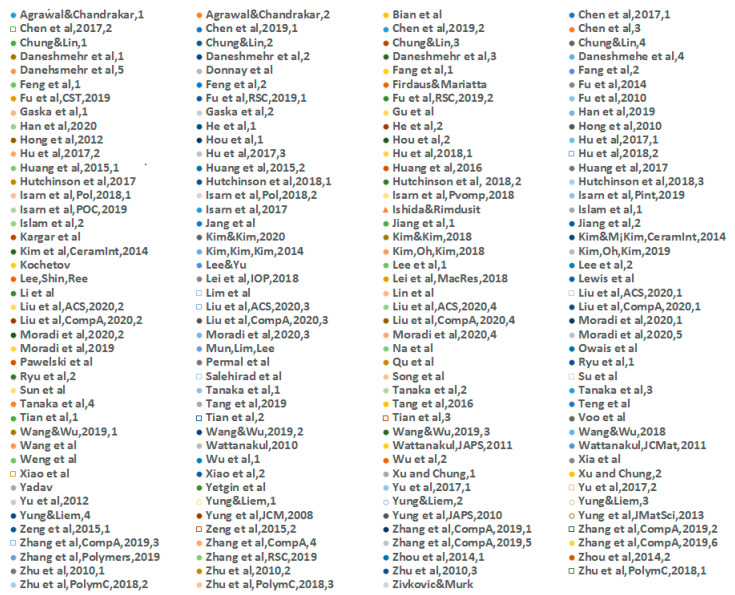
Thermal conductivity of epoxy-BN composites as a function of the BN content by weight. Data taken from references [54,57,59,60,61,62,63,64,65,66,67,68,69,70,71,72,73,74,75,76,77,78,79,80,81,82,83,84,85,86,87,88,89,90,91,92,93,94,95,96,97,98,99,100,101,102,103,104,105,106,107,108,109,110,111,112,113,114,115,116,117,118,119,120,121,122,123,124,125,126,127,128,129,130,131,132,133,134,135,136,137,138,139,140,141,142,143,144,145,146,147,148,149,150,151,152,153,154,155,156,157,158,159]. Open circles refer to data from references [54,151], open squares refer to data from references [89,90,114,116,125,127,132,143,147,153,158]. The dashed trend lines indicate the overall tendency for the dependence of thermal conductivity on BN content (excluding the data represented by the open circles): an upper trend line, below which more than 95% of the values fall; and a lower trend line, below which fewer than 5% of the values fall. The intermediate trend line is an approximation to the upper limit for isotropic samples.

In the foregoing Section 2.2 and Section 2.3, the effect of fillers in general, and of BN in particular, on the cure kinetics of epoxy composites was considered. Although no unequivocal conclusions can be drawn from the results presented in the literature, there is one notable aspect, namely that there is nearly always *some* effect: in the majority of cases, the filler causes an acceleration of the cure, but sometimes there is a retardation. This implies that there is a certain interaction between the filler and the matrix, which might be expected to have some consequences for the properties of the cured composite, and in particular for the thermal conductivity. However, the primary relationship to consider is the effect of BN content on thermal conductivity. It is generally observed not only that the thermal conductivity of epoxy-BN composites increases with BN content, but also that this increase becomes more rapid as the BN content increases, giving rise to an upward curvature in a plot of thermal conductivity as a function of BN content. This upward curvature is usually attributed to increased connectivity between the BN particles beyond the percolation threshold. A compilation of data obtained from the literature is plotted in this way in Figure 7. The relationship between the symbols and the references from which the data were obtained is given in the legend, but can be seen more clearly in Supplementary Material 2.

Included in this compilation are the results obtained for any epoxy composite material containing BN. In particular, many of these references are to hybrids in which BN is incorporated with one or more other type of particle for the enhancement of thermal conductivity. For such hybrid materials, the data plotted in Figure 7 refer only to the limiting situation in which only BN is present. There is a total of approximately 900 data points included in Figure 7, but two series of data are worthy of special mention—the results of Yung and Liem [54] and Yung et al. [151], which are presented as open circles, and which show significantly higher thermal conductivities than any others for the same BN content.

**Figure 8:**
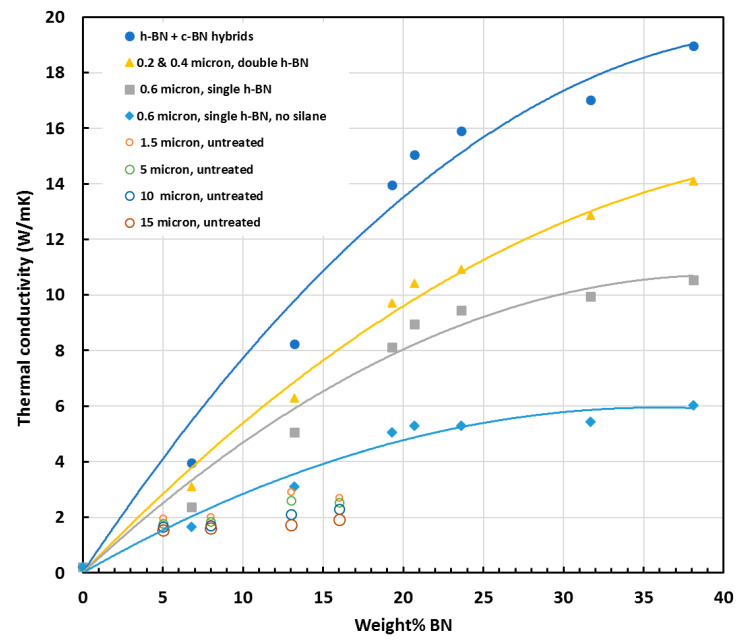
Dependence of thermal conductivity on BN content for epoxy-BN composite systems; data replotted from Yung et al. [54,151]. Filled symbols for silane-treated particles unless otherwise stated [54]: light blue diamonds, 0.6 μm single h-BN, without silane; grey squares, 0.6 μm single h-BN; yellow triangles, 0.2 μm and 0.4 μm double h-BN; dark blue circles, hBN+cBN hybrids. Open symbols [151]: 1.5 μm, 5 μm, 10 μm and 15 μm BN, untreated, symbol size increasing with BN particle size.

In reference [54], the authors use a bisphenol-A type brominated epoxy resin, which has application in flame-retardant printed circuit boards. The epoxy is cured with a dicyandiamide and filled with silane-treated BN particles of different sizes: hexagonal (h-BN), with sizes from about 0.2 μm to 0.6 μm, or cubic (c-BN), with a size of 1.0 μm. In reference [151], the authors use an anhydride-cured bisphenol-A phenolic resin, filled with BN particles of sizes 1.5, 5, 10 and 15 μm, and treated with a silane coupling agent. There is no obvious explanation for why the thermal conductivities are so high. Nevertheless, two aspects are worthy of mention. First, the usually observed upward curvature in the dependence of the thermal conductivity on BN content is not displayed by the results of Yung and Liem [54], which are shown separately in Figure 8; instead, there appears to be a tendency to reach a limiting value as the BN content increases. Second, the results of Yung et al. [151] show a trend of increasing thermal conductivity as the BN particle size decreases, which contrasts with the usual observed effect of particle size [64,65]. This is also illustrated in Figure 8, for the case of the BN particles not treated with silane; the same trend was reported for the silane-treated particles, for which higher thermal conductivities were observed, but for clarity these are not included in Figure 8.

Even setting aside the results of Yung et al. [54,151], there is still evidently a very wide dispersion of the data. In an attempt to clarify the situation, we have included in Figure 7 some trend lines to indicate the overall tendency for the dependence of thermal conductivity on BN content: an upper trend line, below which more than 95% of the values fall (excluding the data of Yung et al. [54,151]); and a lower trend line, below which fewer than 5% of the values fall.

To simplify the picture further, some particular results can be singled out. These are the data which correspond to samples in which orientation of the BN particles has deliberately been introduced. The orientation can be induced by various means: gravity alignment and subsequent resin infiltration [147]; the application of high pressure to partially cured samples before the final cure [90,153,158]; spin-coating of films and hot pressing after pre-cure [116]; magnetic alignment of Fe_3_O_4_-decorated BN particles [114,125,127]; or by resin infiltration of hierarchically ordered 3-dimensional templates [89,132,143]. The orientation induced usually results in a highly anisotropic thermal conductivity. In Figure 7, the results corresponding to the thermal conductivity measured in the orientation direction are shown as open squares, where it can be seen that they almost all lie well above the data for the thermal conductivity of isotropic samples, indicated as the filled data points. Accordingly, we have included a further, intermediate trend line which represents an approximation to the upper limit of these isotropic thermal conductivities. It can be seen that the vast majority of data fall between the lower and intermediate trend lines. The remaining scatter in these data points is a consequence of the many differences in the epoxy-BN composites, which arise from the different parameters involved: the matrix epoxy resin and curing agent; the BN particle size and shape, and the use of hybrids; the use or otherwise of surface treatment of the particles or of coupling agents; and the cure schedule and fabrication procedure. Many of these aspects will be considered below.

Before looking at these aspects, though, there still remain some data points in Figure 7 which have thermal conductivities significantly higher than those indicated by the intermediate trend line, and are worthy of special mention. Ishida and Rimdusit [94] reported, some twenty years ago, “extraordinarily high” thermal conductivities for large BN contents, indicated by the filled triangles in Figure 7, giving the highest value of 32.5 W/mK for 88 wt.% BN. In fact, these authors used a benzoxazine resin and so these results should not strictly be compared with those for epoxy-BN composites, but the very high values for thermal conductivity suggest that there may be some important aspects to consider. There are several points of particular interest. The benzoxazine monomer is solid at room temperature and was first ground to a powder, mixed with the BN particles and heated to about 80 °C, when it took the form of a paste, which was then compression moulded before finally curing under 0.1 MPa pressure at 200 °C. The compression moulding may have introduced an orientation of the BN particles, which would have an important effect on the thermal conductivity. The BN particles were large, with an average size of about 225 μm and a bimodal size distribution; according to the authors, this bimodal distribution facilitated the very high filler contents achieved. Finally, under the composite preparation conditions used, the benzoxazine matrix had a very low viscosity, around 1000 cP, in comparison with that of a typical bisphenol-A epoxy, which is around 12,000 cP; this may have contributed to the ability to obtain very high filler contents. The authors measured the density of the composites and noted that the measured density was slightly higher than that calculated using the densities of the components, which might be expected as a consequence of shrinkage on cure, and they concluded from this that there were no voids in the samples, which is essential for the achievement of high thermal conductivity. Indeed, attempting to add more BN beyond 88 wt.% resulted in a significant decrease in density, which they attributed to void formation. All of these observations are interesting, but do not really explain such high values of thermal conductivity. In fact, the increase in thermal conductivity is dramatic only beyond about 70 wt.%, where the value coincides approximately with the intermediate trend line in Figure 7; for a lower filler content, for example at 50 wt.% BN, the value was unremarkable, and close to the lower trend line. If the dramatic increase is due to the establishment of connectivity between the filler particles, then it suggests that the percolation threshold is much higher than that proposed by the authors, around 20 vol% or 33 wt.%. Nevertheless, the value of 32.5 W/mK for the thermal conductivity remains remarkable, and in 2013 it was stated that it “remains the highest reported thermal conductivity value in the literature” [160].

A value “higher than 30 W/mK” was reported by Song et al. [126] for an epoxy-BN composite with 50 vol% filler, the highest filler content used by these authors and which we estimate to be approximately 65 wt.%. It is important to note here, though, that this value was measured in a thin film, and in the in-plane direction; consequently, there is a strong likelihood that there are significant orientation effects here. It seems reasonable, then, that this value appears to extend, to a slightly higher filler content, the tendency corresponding to the values of the samples in which orientation has been deliberately introduced, discussed above and indicated in Figure 7 by the open squares.

The results discussed immediately above may be considered as special cases, for the reasons presented. There are still two sets of data in particular which stand out from the rest in Figure 7, and which fall between the upper and intermediate trend lines. Both sets of data, indicated by grey-blue and green filled circles, are from Islam et al. [95], and correspond to composites in which the matrix was a liquid crystalline epoxy resin. These results will be discussed in more detail below. The values of thermal conductivity of close to 10 W/mK reported by Tanaka et al. [129] and Xu and Chung [144], indicated in Figure 7 by green and orange circles, respectively, are also worthy of special mention; both use about 70 wt.% BN and the data lie significantly above the intermediate trend line. Nevertheless, the vast majority of the remaining results included in Figure 7 fall between the lower and intermediate trend lines. This still leaves a significant amount of scatter, and the following sections consider the possible reasons for this, and how they might influence the thermal conductivity.

### 3.2. Effect of Surface Treatments and Coupling Agents

It is well known that the interface between the epoxy matrix and the filler particles is a major source of thermal resistance, and for this reason considerable attention has been paid to finding ways in which the quality of this interface can be improved. One problem is that the flat surfaces of the hexagonal BN platelets, which correspond to the hexagonal planes of the BN crystal structure, are molecularly smooth, and present little opportunity for chemical bonding with the matrix or for other interactions. However, there are some functional groups, such as hydroxyls and amino groups, which are present on the narrow edge planes of these platelets, and these edges do offer the possibility of bonding in order to improve the matrix-particle contact. It is, therefore, quite common to aminate [71] or hydroxylate these edges, for example by sol-gel treatment with a strong alkali. These functionalised BN particles may be used directly in the epoxy-BN composite [69,71,85,124], or may additionally be treated with a coupling agent to further enhance the matrix-filler interface. Teng et al. [57] used a zirconate coupling agent, but by far the most common are the silanes, of which numerous variants have been used, including (3-mercaptopropyl) trimethoxysilane, [97], (3-aminopropyl) triethoxysilane, APTES [88,109,138,155], epoxy-terminated dimethyl siloxane, ETDMS [101], and (3-glycidyloxypropyl) trimethoxysilane, GPTMS [66,138]. Jang et al. [96] found that increasing the carbon chain length of the silane, from propyl trimethoxysilane to hexadecyl trimethoxysilane, improved the affinity of BN with epoxy and led to an increase in thermal conductivity. A similar observation was made by Wattanakul et al. [137], who found that increasing the chain length of four cationic surfactants, from dodecyl-, tetradecyl-, hexadecyl- to octadecyl trimethyl ammonium bromide, adsorbed onto the surface of BN particles was increasingly effective for enhancing the thermal conductivity, as a consequence of better wetting of the epoxy resin. In many cases the coupling agent, N-(β-aminoethyl)-γ-aminopropyl trimethoxysilane [54], APTES [68,74,82,131,138,148], or GPTMS [72,73,138,144,156], is added directly in the preparation of the epoxy-BN composite, usually with the help of a solvent such as acetone or ethyl alcohol, and without any prior functionalisation of the BN particles.

Other approaches to the functionalisation of the BN particles involve the use of dopamine [69], 1-pyrenebutyric acid, which reportedly avoids structural defects induced in BN particles by covalently-bonded functional groups [85], ionic liquid flame-retardant functionalisation [113], and aniline trimer, which is considered to provide π-π interactions with the BN particles [124]. Wattanakul et al. [138] also investigated the effect of admicellar polymerisation of polystyrene and polymethylmethacrylate onto the BN surfaces to improve the interfacial adhesion, and found a significant enhancement of the thermal conductivity.

**Figure 9:**
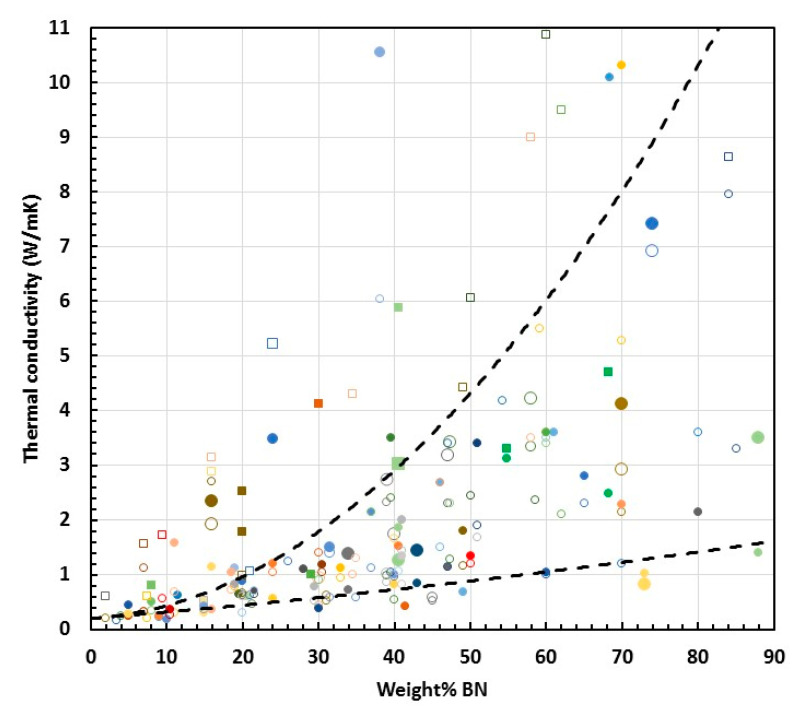

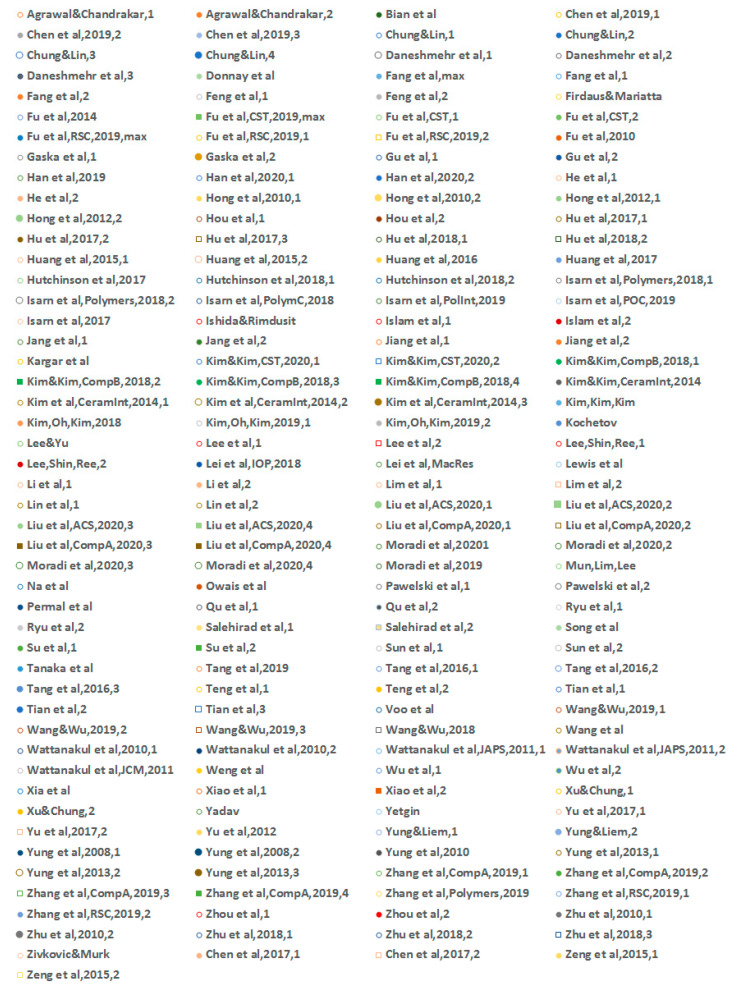
Maximum values of thermal conductivity as a function of BN content for data taken from references [54,57,59,60,61,62,63,64,65,66,67,68,69,70,71,72,73,74,75,76,77,78,79,80,81,82,83,84,85,86,87,88,89,90,91,92,93,94,95,96,97,98,99,100,101,102,103,104,105,106,107,108,109,110,111,112,113,114,115,116,117,118,119,120,121,122,123,124,125,126,127,128,129,130,131,132,133,134,135,136,137,138,139,140,141,142,143,144,145,146,147,148,149,150,151,152,153,154,155,156,157,158,159]. Colour coding for references is given in the legend but can be seen more clearly in Supplementary Material 3. Open circles: untreated particles; filled circles: treated particles. Squares correspond to data from oriented samples. Increasing size of particles within any set of data is represented by increasing size of symbol. Dashed lines are the intermediate and lower trend lines, and are drawn to guide the eye. Some of the data at 20, 30 and 40 wt.% BN have been shifted slightly to lower or higher BN contents to avoid overlapping, for clarity.

Attention was drawn in the previous section to the high value of thermal conductivity—just over 10 W/mK at about 70 wt.% BN—reported by Xu and Chung [144]. It is interesting to note that these authors compared a variety of different surface treatments in the fabrication of epoxy composites, with BN contents of 44 vol% and 57 vol%, which is approximately 58 and 70 wt.%, respectively. In all cases, the thermal conductivity increased with BN content, so we consider here only those with 70 wt.%. The composites with untreated BN particles, equiaxial and from 5 to 11 μm in size, have a thermal conductivity of 5.27 W/mK, which increases sequentially for acetone, nitric acid and sulphuric acid treatments. For the silane treatment, the thermal conductivity increases with increasing proportion of silane, from 0.7% to 2.4%, and at the highest proportion is the most effective treatment, giving a thermal conductivity of 10.31 W/mK. This increase in thermal conductivity of 96% as a consequence of the surface treatment of the BN particles is remarkable, and represents the most dramatic effect of all the surface treatments considered here. This is illustrated in Figure 9, which presents the data of Figure 7 in a slightly different way. First, only the maximum value from each of the references is plotted, rather than showing the data for all BN contents from a given reference source. Second, the scales are limited to 11 W/mK and 90 wt.% BN; in this way, it is possible to show more clearly the region in which most of the experimental data lie and to identify the effects of the parameters, namely the surface treatment and the particle size.

The data of Xu and Chung [144] can be seen at 70 wt.% BN in Figure 9, indicated by filled and open orange circles. A similar increase in thermal conductivity, of almost 75%, when the BN particles were surface treated can be seen in the data of Yung and Liem [54] at 38 wt.% BN, indicated by light blue circles, though the unusual nature of these results was pointed out above. Similarly, a significant increase in thermal conductivity was found by Kim et al. [102] for an epoxy-terminated dimethyl siloxane matrix filled with BN particles which had been treated with either GPTMS or 3-chloropropyl trimethoxysilane (CPTMS). These authors found, for BN contents from 50 to 70 wt.% (indicated in Figure 9 by open and filled brown circles for 70 wt.%), that GPTMS was more effective than CPTMS, although both increased the thermal conductivity when compared with composites fabricated without any coupling agent. In fact, an increase in thermal conductivity as a consequence of surface functionalisation and/or of using a coupling agent is nearly always observed, though the effects are often much smaller than those reported by Xu and Chung and by Yung and Liem. However, there is one report in which the thermal conductivity decreases in nearly all cases after surface treatment [66], and it is argued that for the surface treatment to be effective, the BN particles must be platelets rather than agglomerates, since the latter have fewer edges available for modification. In this respect, it would appear strange that the dramatic increase observed by Xu and Chung [144] should be for what these authors call “equiaxial” particles.

In some instances, the effect of surface treatment is so small as to be virtually negligible [71,74,82,123,131,148,155]. On the other hand, when the thermal conductivity is low (close to the lower trend line in Figure 9), a small absolute increase can translate into a significant percentage increase. This effect can be seen in the results of Lee et al. [109], where for 10 wt.% BN the conductivity increases from 0.27 to 0.36 W/mK after sulphuric acid treatment and silanisation, and in the results of Zhang et al. [155], where it increases from 0.37 to 0.43 W/mK at 15 wt.% BN after oxidisation in air and the addition of APTES. In contrast, again at a low BN content of only 10 wt.%, the non-covalent functionalisation with pyrenebutyric acid by He et al. [85] results in a remarkable increase in thermal conductivity from 0.68 to 1.58 W/mK, well above the intermediate trend line.

There are some other results which should be commented on. Chung and Lin [72] achieved high BN content, but found that the thermal conductivity passes through a maximum at 50 to 60 vol% for two different BN particle sizes, 3.6 and 10.6 μm, the latter exhibiting higher thermal conductivity. When the GPTMS coupling agent was used without prior functionalisation of the BN particles, the maximum thermal conductivity increased by about 0.5 W/mK for each size of particle, leading to a value of 7.42 W/mK for the larger particle, which is approaching the intermediate trend line in Figure 9. Jang et al. [96] observed an increase from 2.4 to nearly 3.5 W/mK for a BN content of close to 40 wt.%, which represents a move from below to well above the intermediate trend line; this was for BN which had been surface modified by a sol-gel reaction with NH_4_OH and a silane coupling agent with a C16 carbon main chain. Similarly, Wattanakul et al. [137,138] investigated various strategies, including the use of admicellar polymerisation modification of the BN surfaces and the use of cationic surfactants of different chain length. These treatments resulted in significant increases in thermal conductivity, with maximum values of 3.4 W/mK at 51 wt.% BN and 2.69 W/mK at 46 wt.% BN, both of which are close to the intermediate trend line.

Other results for which thermal conductivity values fall close to the intermediate trend line should also be mentioned. Jiang et al. [97] used a strong alkali to attach –OH groups to the BN particles and then grafted the mercaptopropyltrimethoxysilane coupling agent. The increase in thermal conductivity as a consequence of the surface treatment resulted in a maximum value of 1.2 W/mK, which is relatively high for a BN content of only 24 wt.%. Likewise, Li et al. [113] achieved a relatively high value of 1.04 W/mK for only 20 wt.% BN in a flame-retardant epoxy-BN nanocomposite. At slightly higher BN contents, Tang et al. [131] achieved a value of 1.51 W/mK at 30 wt.% BN with APTES as the coupling agent.

It is evident that in virtually all cases the surface treatment of the BN particles and/or the use of coupling agents, in all their varieties, result in an enhancement of thermal conductivity. However, this comes at a certain cost in many instances. One of the important parameters with respect to the use of these epoxy-BN composites in insulated metal substrates is the simplicity of the fabrication process, as mentioned in the Introduction, but many of the results discussed above make use of a solvent in the dispersion of the functionalised BN particles in the epoxy resin system, which is generally an undesired industrial practice. The solvents most widely used are acetone [57,74,85,113,131,141,148] and ethanol [68,82,97,155], but others include methyl ethyl ketone and dimethyl formamide [96], and tetrahydrofuran [71]. On the other hand, “dry” or “direct” mixing, which obviates the use of any solvents in this composite fabrication step, was adopted by many researchers [54,88,109,113,123,137,138,144,149,156]. Chung and Lin [72] used dry mixing for BN contents ≤40 vol% but required acetone as a solvent for dispersion in order to achieve higher filler loadings.

While there seem to be clear advantages in the use of surface treatment to enhance thermal conductivity, it should be pointed out that there are several researchers who did not make use of such treatments yet achieved notable values of thermal conductivity. These results, represented by open circles in Figure 9 and which lie close to the intermediate trend line, are worthy of some comment. Gaska et al. [81] used a DGEBA epoxy cured with anhydride, and reported thermal conductivity values of 2.32 W/mK and 2.75 W/mK at a filler loading of 25 vol% (approximately 39 wt.% BN), for untreated BN particles with average sizes of 13 μm and 25 μm, respectively. The effect of particle size will be considered later, but it is remarkable that such values of thermal conductivity are achieved with simple mixing with untreated BN particles. Approximately the same value of thermal conductivity as that for the smaller particles above was reported by Jang et al. [96], namely 2.40 W/mK at approximately 39 wt.% BN, for untreated platelets with sizes in the range of from 5 to 10 μm; the unspecified epoxy resin was cured with dicyandiamide, but the composite preparation required the use of methyl ethyl ketone and dimethylformamide.

At slightly higher BN contents, Hutchinson et al. [64,65,66] reported values from 3.2 to 3.4 W/mK at about 47 wt.% BN for particles of various sizes and types, and values of approximately 4.2 W/mK at 54 wt.% and 58 wt.% BN. These values, higher than the majority of other values reported in the literature, both with and without surface treatment, were all obtained with untreated BN particles, and with a DGEBA epoxy resin matrix cured with a thiol, PETMP. These authors suggested that the Lewis acid-base interaction between the sulphur in the thiol and the boron in the filler leads to an improved matrix-filler interface, which was the reason for the enhanced thermal conductivity. In support of this hypothesis, they reported that the same epoxy-BN system cured with a diamine does not give such high thermal conductivities, for example only 1.7 W/mK at 53 wt.% BN [65]. They also noted that the cure kinetics of the epoxy-thiol system and the epoxy-diamine system are quite different in their dependence on the BN content, as discussed in Section 2, and consider this to be further evidence of the favourable matrix-filler interaction in the epoxy-thiol-BN composite system.

The result obtained by Kargar et al. [98] is also noteworthy. These researchers used an unspecified epoxy and curing agent and obtained high volume fractions of untreated BN platelets, with sizes in the range from 3 to 8 μm, without the use of solvents. The reported thermal conductivity of 5.5 W/mK at a filler loading of 59 wt.% (open yellow circle) is very close to the intermediate trend line in Figure 9.

### 3.3. Effect of BN Particle Size and Shape

Another parameter that is of prime importance in respect of the thermal conductivity of epoxy-BN composites is the size and shape of the particles used as the filler. The interface between filler particles and matrix is important because it represents a thermal barrier in the pathway for phonon transport between the highly conducting BN particles. There will inevitably be some heat conduction through the epoxy resin between the filler particles, and it is for this reason that much effort has been expended in attempting to minimise the thermal barrier of the interface by surface treatments of the BN particles and by the use of coupling agents, as discussed in the previous section. For a given filler loading, however, the amount of interfacial region depends on the size of the filler particles, which decreases as the particle size increases. Consequently, one might expect that the thermal conductivity of an epoxy-BN composite with a given weight fraction of filler would be greater the larger the filler particles are. We examine this idea by referring to the data collected in the literature.

In Figure 9, the results obtained for a given system in which BN particles of different sizes are used are presented such that the size of the data point increases with increasing particle size. In nearly all cases, thermal conductivity increases as the particle size increases, as expected. For Chung and Lin [72], who also investigated the effects of using a coupling agent, this increase was quite dramatic, going from about 2.3 to 7.0 W/mK with no coupling agent, and from about 2.8 to 7.5 W/mK with a coupling agent, when the average particle size increased from 3.6 to 10.6 μm (dark blue circles, open and filled, at 65 and 74 wt.%). Hong et al. [87] investigated the effect of a bimodal distribution of polygonal aluminium nitride particles and platelet-shaped BN particles, with a view to increasing the packing efficiency and maximum filler content, but also reported the thermal conductivity of highly filled composites containing only BN particles, with average sizes of 6 μm and 18 μm. For a very high content of 70 vol%, which approximates to 88 wt.% BN, and with APTES as the coupling agent, the thermal conductivity increased from 1.4 to 3.5 W/mK with increasing particle size. Although these values of thermal conductivity are low for such a high filler content, as is evident from Figure 9 (filled light green circles), they display the usual dependence on particle size. The slight decrease in thermal conductivity at 73 wt.% BN with increasing particle size, for values taken from earlier work by the same group [86] (filled yellow circles), is misleading. In this earlier work, the authors were studying the effect of different quantities of the APTES coupling agent; the maximum thermal conductivity occurred for different silane contents for the two particle sizes (1 and 5 μm) and the variation in thermal conductivity was small, so this result is not likely to be significant.

Kim et al. [102], who investigated the effect of different coupling agents, as discussed in the previous section, also studied the effect of different sizes of BN particles, namely 1, 8 and 12 μm, and observed a systematic increase in the thermal conductivity with increasing particle size for each of their filler contents of 50, 60 and 70 wt.%. The result for 70 wt.% BN is included in Figure 9 (open brown circles), where it can be seen that the thermal conductivity was rather low for such a high filler content, though the use of the coupling agent improved it (filled brown circle).

There are several results where the thermal conductivity for composites with a given filler content lies close to the lower trend line in Figure 9, but with an increase in the particle size the thermal conductivity increases, sometimes even approaching the intermediate trend line. Isarn et al. [61], in their studies of composites with a cycloaliphatic epoxy cured with a thiol, obtained a value close to 1.0 W/mK for 40 wt.% of 6 μm particles, which increased significantly to 1.7 W/mK for the same loading of 80 μm particles, a value mid-way between the lower and intermediate trend lines. In another example, Tang et al. [131] obtained a thermal conductivity of 0.6 W/mK at a filler loading of 18 vol% (approximately 30 wt.%) for 1.5 μm particles, which lies on the lower trend line. This value increased to 1.4 W/mK for 30 μm particles (open blue circles, displaced to 31 wt.% for clarity), a value close to the intermediate trend line. Furthermore, this value increased slightly when a coupling agent was used (filled blue circle). Similar results were reported by Yung et al. [149] with a particle size increase from 53 nm to 4 μm for 43 wt.% BN, and by Zhu et al. [157] with a particle size increase from 70 nm to 7 μm for 34 wt.% BN. The same effect of increasing thermal conductivity with increasing particle size is therefore also seen in the size range from nanometre to micrometre.

While the majority of researchers have found that thermal conductivity increases with BN particle size, there are some, though rather few, reports of almost no effect, or even a decrease. Pawelski et al. [121] used a Novolac epoxy matrix cured with diethylmethyl benzenediamine and filled with 45 wt.% BN particles 2, 12 and 45 μm in size, all in the form of platelets. The thermal conductivities measured were 0.53, 0.59 and 0.66 W/mK, respectively, thus increasing systematically with particle size, but very little when viewed in the context of other values reported in the literature. This is seen clearly in Figure 9, where these values are all clustered just below the lower trend line (open purple circles). Likewise, Permal et al. [122] observed no effect of particle size, for BN platelets with average sizes of 1 and 5 μm, in their composites with 30 wt.% filler. They used an epoxy matrix that was a mixture of DGEBA, Novolac and cycloaliphatic epoxy, cured with anhydride. Similar to the study by Pawelski et al., the value of the thermal conductivity was very low for the filler content used, and lies just below the lower trend line in Figure 9 (dark blue filled circle).

The notable report of a decrease in thermal conductivity as BN particles size increases can be found in the work of Yung et al. [151]. These authors investigated epoxy-BN composites with a bisphenol-A phenolic matrix cured with an anhydride and filled with platelets with sizes ranging from 1.5 to 15 μm, both with and without a silane coupling agent. The use of the silane coupling agent resulted in an increase of thermal conductivity, but there was a decrease in thermal conductivity for increasing particle size, typically by between 20% and 30%, whether or not the coupling agent was used, and for all the filler contents used, from 3 to 10 vol%, equating to about 5 to 16 wt.%. These data can be seen to fall well above the intermediate trend line in Figure 9 at 16 wt.% BN (open and filled brown circles), and even above the upper trend line in Figure 7. The fact that these authors found trends of thermal conductivity as a function of BN content and particle size that are opposite to nearly all other reports in the literature suggests that there may be some other factors at play here, such as orientation or sedimentation of the particles during the fabrication of the composites.

Finally, it is important to point out that it is not always straightforward to deduce the effect of particle size from results presented in the literature, as there is a simultaneous effect of particle shape which can complicate the situation. For example, Gaska et al. [81], in a study of epoxy composites filled with hybrids of aluminium nitride, boron nitride and silica, compared the thermal conductivity of two epoxy-BN composites, one filled with platelet-shaped particles with an average size of 13 μm and the other filled with agglomerates with an average size of 25 μm. The thermal conductivity of composites filled with 25 vol% (approximately 39 wt.%) of the larger agglomerate particles was 2.75 W/mK, falling on the intermediate trend line in Figure 9, in comparison with only 2.32 W/mK for the same content of the smaller platelet particles (open light brown circles). This seems to accord with the usually observed increase in thermal conductivity with increasing particle size, but this conclusion does not take into consideration the effect of the shape of the particles.

Likewise, Huang et al. [91] used BN fillers in different forms, namely spherical particles with a narrow size distribution from 200 to 400 nm, and “flakes” with a diameter from 3 to 6 μm and a thickness from 100 to 300 nm. These authors found that, for filler loadings up to 30 wt.%, the thermal conductivity was 0.95 W/mK for the composites fabricated with flakes compared with 0.45 W/mK for the composites fabricated with the smaller spherical particles. Both of these values are rather low for the filler content used, the latter falling on the lower trend line. Again, though, the particles with the larger diameter give a higher thermal conductivity, but in this case, in contrast to the findings of Gaska et al., it is the platelet-shaped particles which had the higher conductivity.

Similar to Gaska et al., Sun et al. [128] compared composites fabricated with up to 40 wt.% of larger (30 μm) spherical particles with those fabricated with smaller (18 μm) platelets and found that the thermal conductivity was higher for the larger spherical particles. For example, at 40 wt.% the thermal conductivity was 1.03 W/mK for the spherical particles in comparison with 0.86 W/mK for the platelets. These values fall well below those found by Gaska et al. for particles of similar sizes, and in fact are only slightly above the lower trend line in Figure 9, but nevertheless show this same trend of increasing thermal conductivity with particle size. In fact, the preparation procedure for these composites involves a hot pressing stage; this induces a certain amount of in-plane orientation for the platelets, which does not occur for the equiaxial spherical particles. The values quoted above correspond to the through-plane direction, and hence disadvantage the platelets, for which the through-plane thermal conductivity of the platelets themselves (2 W/mK according to Sun et al.) is much smaller than the in-plane value (185 to 300 W/mK according to Sun et al.). This effect of orientation will be considered later, but is clearly a further complication in the analysis of the effect of particle size.

The findings of Gaska et al. [81], Huang et al. [91] and Sun et al. [128] demonstrate an increase of thermal conductivity with increasing particle size, but it is not possible to draw any unequivocal conclusions from them about the effect of particle size, as there are other intervening factors. On the other hand, Hutchinson and co-workers [63,64,65,66,67] conducted a systematic study of the effects of both BN particle size and shape. Moradi et al. [65] fabricated epoxy-thiol composites filled with BN particles of different average sizes, namely 2, 30 and 180 μm, all in the form of platelets, and found an increase of thermal conductivity with increasing particle size for a given filler content. For example, for 47 wt.% BN the thermal conductivity increased from 1.28 W/mK for the 2 μm particles to 2.36 W/mK for the 30 μm particles and reached 3.02 W/mK for the 180 μm particles. In Figure 9, this can be seen to represent an increase from just above the lower trend line for the smallest particles to slightly below the intermediate trend line for the largest particles (open dark green circles). This filler content of 47 wt.% was the limiting amount for the 2 μm platelets, but higher contents were achieved for the larger particles, with the thermal conductivity reaching a value of 4.22 W/mK at 58 wt.% filler content; this value can also be seen in Figure 9 to be approaching the intermediate trend line. The same effect of particle size was reported also by this group for agglomerates: for example, at 47 wt.% filler content, the thermal conductivity for composites made with lightly agglomerated 6 μm particles was 2.31 W/mK [63], whereas a significantly higher value of 3.40 W/mK was measured when 80 μm agglomerates were used [64]. It is interesting to note that with the use of hybrids, in which BN particles of different sizes are incorporated simultaneously, in particular 80/6 μm and 80/2 μm [64], the thermal conductivity did not increase for a given filler content, but decreased. This result is consistent with larger particles giving higher thermal conductivity. What the hybrids do permit, on the other hand, is a higher packing density, and hence higher thermal conductivities were achieved as a consequence of higher filler contents [64]. This same group also compared the effects of particles of different shapes, namely platelets and agglomerates [67], and found that agglomerates are more effective in enhancing the thermal conductivity. For example, for all the filler contents investigated, 120 μm agglomerates gave a higher thermal conductivity than the larger 180 μm platelets.

The reason why larger particles should result in higher thermal conductivities for a given filler content is probably related to the effect of the matrix-particle interface, which represents a certain barrier to heat transfer. It is for this reason that much attention has been paid to improving this interface, as discussed in Section 3.2. For a given filler content, this interfacial area is smaller for larger filler particles, and hence one would expect the effect of particle size that is generally observed. However, increasing particle size has its limitations as a strategy for increasing the thermal conductivity of insulated metal substrates; in practice, the dielectric layer is usually between 50 and 150 μm in thickness, and filler sizes of less than about 30 μm are preferred.

## 4. Special Procedures

### 4.1. Orientation

The quest for higher values of thermal conductivity of epoxy-BN composites has encouraged a large number of researchers to investigate novel fabrication procedures in recent years. In many cases these procedures involve orientation of the BN platelets in some way or another in order to benefit from the much higher in-plane thermal conductivity of the BN platelets themselves in comparison with the through-plane direction. In general, these specialised fabrication procedures create a structured assembly for which there is a highly anisotropic thermal conductivity and often a significant enhancement of thermal conductivity. This can be seen clearly in Figure 7, where the open squares represent data obtained from composites fabricated by such procedures. Many of these data points fall close to the upper trend line. Here, we review some of these special procedures.

Orientation of the filler particles can be achieved in a number of ways, possibly even simply by sedimentation of the particles in a low viscosity mixture. Kim and Kim [100] fabricated composites with an epoxy-terminated dimethyl siloxane matrix cured with diaminodiphenylmethane, in which the BN particles, either as 12 μm h-BN platelets or exfoliated BN nanosheets (BNNS), were incorporated in solution in ethanol. During solvent removal by filtration, the larger h-BN particles were preferentially oriented to a greater extent than the BNNS, which resulted in an anisotropic thermal conductivity, with higher values and a greater degree of anisotropy for the former. For a composite with 68 wt.% h-BN, the thermal conductivity increased from 2.5 W/mK in the through-plane direction to 4.7 W/mK in the in-plane direction, as can be seen in Figure 9 (filled dark green symbols). In a slightly different procedure, orientation was also induced by gravity alignment of 10 to 30 μm size h-BN platelets by Yu et al. [147] during vacuum filtration from an ethanol solution to form a h-BN “cake”, in the form of a cylinder of diameter 4 cm and thickness 3 cm, in which the BN platelets were oriented parallel to the base of the cylinder. This cake was then sliced thinly, with the BN platelets perpendicular to the plane of the slices, infiltrated with an acetone solution of DGEBA epoxy resin, hexahydro-4-methylphthalic anhydride curing agent and dimethylaminomethylphenol accelerator, degassed under vacuum, and cured at 100 °C for 2 h. For 44 vol% BN content, this significant amount of orientation gave a thermal conductivity in the through-plane direction of 9.0 W/mK in comparison with 3.5 W/mK for a simple random dispersion of the same BN content in the epoxy system. These results are included in Figure 9 at an estimated 58 wt.% BN (open dark pink symbols), where it can be seen that the orientation increases the thermal conductivity from approximately mid-way between the lower and intermediate trend lines to well above the intermediate trend line; nevertheless, Figure 7 shows that the oriented sample still lies significantly below the upper trend line.

Filtration through a die was also the technique adopted by Xiao et al. [143] to induce orientation of BNNS in composites. They used a more elaborate fabrication procedure, in which the BNNS were first exfoliated from 12 μm BN platelets by sonication in isopropyl alcohol and were then both functionalised with GPTMS and decorated with SiC nanowires (SiC_w_, 0.1 to 0.6 μm in diameter and 50 to 100 μm long) in an ethanol solution before being vacuum filtrated through a die. They achieved a vertically-oriented SiC_w_/BNNS framework which was vacuum-assisted infiltrated with epoxy resin and anhydride curing agent, and finally cured. Samples were prepared with different proportions of SiC_w_ and BNNS, and while the thermal conductivity increased with BNNS content as usual, for a given filler content the thermal conductivity increased with increasing proportion of BNNS, leading us to question why the SiC_w_ was incorporated. The highest thermal conductivity obtained was 4.22 W/mK in the direction of orientation for a sample containing 21.9 vol% filler (approximately 30 wt.%) with a 1:9 mass ratio of SiC_w_ and BNNS. The corresponding value in the orthogonal direction was 1.43 W/mK, and these values can be compared with 1.6 W/mK for a randomly distributed epoxy/BNNS composite. The thermal conductivity of the oriented sample lies on the upper trend line in Figure 7 and well above the intermediate trend line in Figure 9 (filled maroon square), and hence represents a significant result. In fact, this result is quite similar to those obtained by Yu et al. [147], Hu et al. [90], Liu et al. [116], and Lim et al. [114]. All these values of thermal conductivity, at different filler contents for each of these groups, increased as a consequence of the various methods of orientation, from a value approximately mid-way between the lower and intermediate trend lines to a value well above the intermediate trend line.

Similarly, Lee et al. [108] observed a significant increase, from 0.6 W/mK in the through-plane direction to 1.7 W/mK in the in-plane direction, for composites with only 10 wt.% BN. This is again clearly seen in Figure 9 (open red symbols). The fabrication procedure involved the use of a solvent, acetone, to incorporate the 1.5 to 2.0 μm BN particles into the epoxy-diamine mixture, and the low viscosity and low filler loading probably resulted in sedimentation, though this is not specifically stated by the authors.

**Figure 10:**
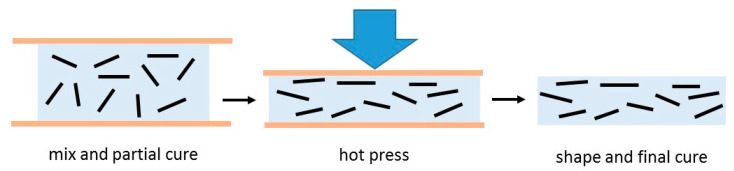
Schematic illustration of the hot-pressing procedure used to introduce orientation into epoxy-BN composites.

Perhaps a more direct way in which to induce orientation, and one which has been used by a number of researchers, is by hot-pressing. This is well illustrated by the results of Hu et al. [90] on composites with an anhydride-cured epoxy matrix and 18 μm BN particles. In the first stage of the fabrication procedure, illustrated in Figure 10, the composite was mixed and partially cured for 15 min at 120 °C to provide sufficient viscosity for the subsequent stage, in which a pressure of 10 MPa at 150 °C was applied to the composite sample between copper foils. The hot-pressed composite was then moulded into a disc shape with diameter 25.4 mm and thickness 200 μm, and finally cured at temperatures from 120 °C to 200 °C for programmed times. The particle orientation induced by the hot-pressing was detected by X-ray diffraction (XRD), and the degree of orientation was found to increase as the filler loading increased from 40 to 60 wt.%. The anisotropy induced at 50 wt.% was notable: 6.09 W/mK for the oriented sample in comparison with 2.44 W/mK for a sample with random orientation of the BN particles. In Figure 9, this orientation effect can be seen to increase the thermal conductivity from well below the intermediate trend line to well above it (open black symbols). A more dramatic increase, to 10.87 W/mK, was found for the higher filler loading of 60 wt.%, as can be seen in Figure 9, but the increased viscosity of this mixture required the use of acetone as a solvent. In fact, these authors carried out a systematic study including different temperatures (130, 150 and 170 °C) and pressures (7, 10, 13 MPa) for the pre-curing stage, as well as different particle sizes of 5, 18 and 25 μm. In this respect, it is interesting to note that increasing pressure results in higher thermal conductivity, as does increasing the size of the BN particles.

Similar results were reported by Liu et al. [116] for a low viscosity epoxy-amine filled system using isopropanol as a solvent, with either 1 to 2 μm h-BN platelets or exfoliated BNNS of about 300 nm lateral dimension. The fabrication procedure of composite films involved spin-coating followed by evaporation of the solvent, and then pre-curing for 5 h at room temperature to provide the necessary viscosity of the mixture. This mixture was then cured under pressure at 5 MPa and 80 °C for 30 min before completing the cure over 5 h at 60 °C. The 40 wt.% BN composites showed considerable anisotropy, more so for the BNNS than for the h-BN. For example, in the through-plane direction the thermal conductivity was 1.9 W/mK for the BNNS composite and 1.3 W/mK for the h-BN composite, which increased to 6.0 W/mK and 3.0 W/mK in the in-plane direction, respectively; in Figure 9, these in-plane values lie well above and just on the intermediate trend line, respectively (filled sage green squares). The greater thermal conductivity for the BNNS filler was attributed to a reduction, by an order of magnitude, in the interfacial thermal resistance between filler and matrix in comparison with the h-BN filler, as a consequence of the use of a surface coupling agent (APTES).

An even more dramatic enhancement of thermal conductivity by orientation was reported by Zhang et al. [153]. These authors investigated an anhydride-cured bisphenol-A epoxy system in which the 18 μm BN platelets were decorated with Ag nanoparticles, an approach that will be further discussed in Section 4.3. The fabrication procedure included the use of hot-pressing, and the effect of this can be considered separately from that of the Ag nanoparticles as the authors used a control sample with undecorated particles, prepared following the same procedure of hot-pressing (“Hot-pressed BN/epoxy”), as well as a sample prepared without hot-pressing (“Random BN/epoxy”). The hot-pressing was made under a relatively low pressure of 30 psi (≈200 kPa) at 100 °C for 10 min, followed by a final cure schedule at temperatures up to 200 °C for programmed times. For a filler content of 62 wt.%, the random BN/epoxy sample had the same thermal conductivity of 2.1 W/mK in both the in-plane and through-plane directions, as would be expected, while the hot-pressed BN/epoxy sample had a thermal conductivity of 9.5 W/mK in the in-plane direction compared with 2.6 W/mK in the through-plane direction. This orientation can be seen in Figure 9 (open dark green symbols) to increase the thermal conductivity from somewhat above the lower trend line to well above the intermediate trend line, even at this relatively low pressure.

Much higher pressures and filler contents were used by Zhu et al. [158], but there seems to be very little effect of the magnitude of the pressure on thermal conductivity. In some way this is consistent with the observation by Zhang et al. [153] that a low pressure can induce significant orientation and hence dramatically enhance thermal conductivity. Zhu et al. fabricated their composites by incorporating their BN platelets into an amine-cured epoxy using tetrahydrofuran as solvent, and compressed their samples at pressures of 43, 108 and 215 MPa for 5 min in a 24 mm diameter mould before curing them in an oven at 80 °C for 30 min and then at 120 °C for 60 min. It should be noted that this procedure is significantly different from the hot-pressing used by Hu et al. [90], Liu et al. [116] and Zhang et al. [153], in which the pre-cure took place under pressure. From SEM images of fracture surfaces of the cured composites, Zhu et al. concluded that there is a certain orientation of the BN platelets as a consequence of the compression process, but that this orientation is not significantly affected by the magnitude of the pressure applied. In contrast, for samples prepared without the application of pressure there was no observable orientation. This lack of dependence of orientation on the magnitude of the pressure is reflected in the thermal conductivity; although there is a fair amount of scatter in the data, there does not appear to be any systematic or significant dependence on pressure, but the thermal conductivity is significantly higher than that for samples prepared without pressure. For example, at 84 wt.% BN and 215 MPa pressure, the through-plane and in-plane thermal conductivities were 7.95 W/mK and 8.63 W/mK, respectively, in comparison with only 3.3 W/mK for the sample prepared without compression. This effect of pressure can be seen very clearly in Figure 9 (open navy blue symbols). The rather small difference between the through-plane and in-plane thermal conductivities for a filler content of 84 wt.% is perhaps surprising, but it becomes much more marked for higher filler contents, and a thermal conductivity of over 20 W/mK was achieved in the in-plane direction at filler contents of about 90 wt.%.

An alternative way in which orientation can be introduced into epoxy-BN composite systems is by magnetic alignment, an approach that has been used by several researchers, most notably Lim et al. [114] and Kim and Kim [99], both groups following similar procedures for decorating the BN platelets with Fe_3_O_4_ nanoparticles. In order to obtain the desired orientation, the uncured epoxy-BN-Fe_3_O_4_ mixtures were placed in a magnetic field before curing. Lim et al. [114] cast 1 mm thick films with 20 vol% (≈34 wt.%) of 8 μm BN platelets from an epoxy solution in methylethylketone to obtain a vertical orientation of the platelets with respect to the plane of the film. Without magnetic orientation, the thermal conductivity was less than 1.0 W/mK, whereas for the oriented film it was 4.3 ± 0.5 W/mK in the vertical direction, thus increasing from just above the lower trend line in Figure 9 to close to the upper trend line in Figure 7. More recently, Kim and Kim [99] used Fe_3_O_4_-decorated BN nanoplatelets, which had previously been liquid phase exfoliated by ultrasonication from 12 μm h-BN particles in dimethylformamide. These decorated particles were then dispersed in proportions up to 20 wt.% in a DGEBA epoxy/acetone solution, before removal of the acetone and addition of the diaminodiphenylmethane curing agent. The doping of the surface of the BN nanoplatelets with Fe_3_O_4_ was confirmed by XRD and X-ray photoelectron spectroscopy (XPS), while the orientation of the nanoplatelets was also confirmed by XRD. Compared with composites fabricated with the raw h-BN particles, for which the thermal conductivity at 20 wt.% was 0.62 W/mK, the same content of the Fe_3_O_4_-doped nanoplatelets gave a slightly lower value of 0.48 W/mK without alignment, but a higher value of 1.07 W/mK with magnetic alignment. This last value falls just on the intermediate trend line and so is somewhat disappointing, while the lower value for the unaligned composite was attributed to the effect of additional Kapitza resistance introduced by the Fe_3_O_4_ nanoparticles.

In terms of enhancing thermal conductivity by the magnetic orientation procedure, the recent results reported by Kim and Kim [99] do not compare very favourably with the earlier work of Lim et al. [114]. Some other relatively recent results were also less successful. Salehirad et al. [125] adopted the same magnetic alignment principle but used a slightly modified procedure in which 70 nm h-BN nanoparticles were first exfoliated into nanosheets by sonication, polyacrylamide was then grafted and the nanosheets were finally modified with Fe_3_O_4_ nanoparticles. Epoxy composites with filler loadings up to 20 wt.% were cast in a mould located between two magnets, but the results were not definitive. Indeed, at the filler content of 20 wt.%, the highest thermal conductivity was found for the polyacrylamide-grafted BN nanosheets without Fe_3_O_4_ modification, while there was no systematic difference in thermal conductivity between the two orthogonal directions after orientation. The lower values found for the Fe_3_O_4_-modified nanosheets are possibly again due to increased Kapitza resistance, while the highest thermal conductivity of only about 0.4 W/mK at 20 wt.% is very close to the lower trend line in Figure 9. Similarly, the results of Su et al. [127] for flexible cycloaliphatic epoxy-BN composites are rather disappointing. Again, there is essentially no significant difference between in-plane and through-plane thermal conductivity after magnetic alignment, and the maximum value of thermal conductivity of about 1.0 W/mK at 30 wt.% filler content lies only mid-way between the lower and intermediate trend lines in Figure 9.

### 4.2. Three-Dimensional Structures

The basic objective of inducing orientation in composites by means of hot-pressing or magnetic alignment is to create preferential pathways for heat transport. In recent years, the same objective of creating pathways for phonon transport has been met in a number of ingenious ways, which rely essentially on the construction of a three-dimensional framework into which the BN particles can be introduced in an ordered way. In most cases, a further aim is to obtain high thermal conductivity at low filler loadings, for reasons of processability, and consequently many of the examples presented here use relatively low filler contents.

**Figure 11:**
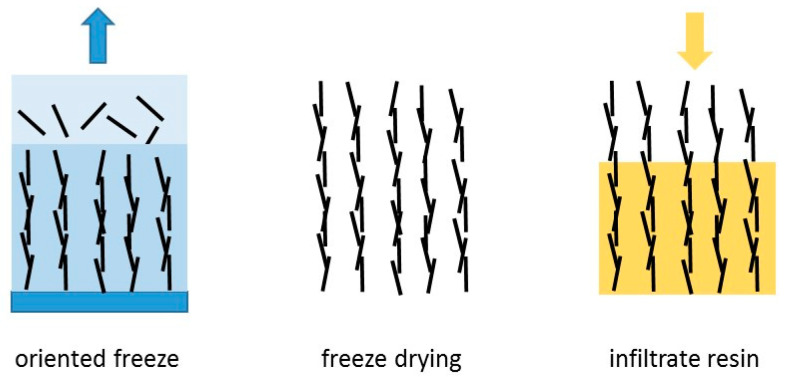
Schematic illustration of aerogel procedure for fabricating 3-dimensional oriented epoxy- BN nanosheets (BNNS) composites.

One quite common procedure is to create an aerogel-type structure with a vertically aligned anisotropic 3-dimensional BNNS network into which the epoxy resin system is infiltrated, as illustrated schematically in Figure 11. The aerogel fabrication often [70,89,134,152], but not always [79], makes use of the ice-templated assembly method, and the skeleton support can be constructed with materials other than BNNS such as cellulose [70] or polyamide 6,6 [79]. In these aerogel-type epoxy-BN nanocomposites, the thermal conductivity is nearly always measured only in the alignment direction, but Zeng et al. [152] compared the parallel and perpendicular directions. They found that the parallel direction gave significantly higher values of thermal conductivity at very low loadings, from 2 to 4 vol%, but that at higher loadings of around 10 vol% there was rather little difference, which they attributed to a microstructural change from a well-ordered wall-like to a honeycomb-like morphology. At 9.29 vol% BNNS, for example, the thermal conductivities in the parallel and perpendicular directions were 2.85 W/mK and 2.40 W/mK, respectively, compared with 1.13 W/mK for a composite with randomly distributed BNNS and with 0.16 W/mK for the epoxy alone. For a similar BNNS content of 9.6 vol%, Chen et al. [70] reported a thermal conductivity of 3.13 W/mK. The BNNS, with a lateral size of around 1.3 μm and thickness of about 2.5 nm, and previously exfoliated from h-BN particles, were incorporated into a cellulose supporting structure, which was infiltrated with a cycloaliphatic epoxy resin with methyl-hexahydrophthalic anhydride as a curing agent and neodymium acetylacetonate trihydrate as a latent catalyst. These values of thermal conductivity obtained by Zeng et al. [152] and Chen et al. [70] are remarkable for such a low filler content. If the 9 vol% content is approximated as 15 wt.% based on the densities of BN and epoxy, it can be seen from Figure 7 that they fall well above the upper trend line.

At even lower filler contents, this aerogel procedure also produces some impressive thermal conductivity results. Wang and Wu [134] infiltrated bisphenol-F epoxy and *o*-dichloroaniline methane as curing agent under vacuum into the vertically aligned 3-dimensional BNNS network and measured a value of 1.56 W/mK in the cured composite with only 4.4 vol% filler. This was significantly higher than the value of 0.32 W/mK for the same content of randomly dispersed BNNS. If the filler content is approximated as 8 wt.%, this result can be seen to lie well above the intermediate trend line in Figure 9 (open maroon symbols), and just above the upper trend line in Figure 7. On the other hand, the results reported by Fu et al. [79] for a filler content of 4 vol% are not quite so impressive. These workers prepared a polyamide 6,6 aerogel structure, to which the BN platelets, with sizes between 3 and 5 μm, adhered and formed a continuous thermal conduction network. After infusion of the epoxy resin and diaminodiphenyl methane curing agent, and subsequent curing of the composite, they measured a value of 0.6 W/mK. Although this is three times higher than the thermal conductivity of a composite with the same content of a random dispersion of the same BN platelets, it lies only just above the intermediate trend line in Figure 9 (open yellow square).

In contrast to the low filler contents used in the works described above, Hu et al. [89] introduced large quantities of filler into their three-dimensionally structured composites. Their aerogel was prepared by ice-templating and then freeze-drying an aqueous slurry of 10 μm h-BN platelets with sodium carboxymethyl cellulose, which acts as both a dispersion agent and organic binder. The epoxy resin, anhydride curing agent and imidazole catalyst were mixed and infused into the aerogel, and the composite was finally cured at 120 °C for 1 h and then at 160 °C for 2 h. The thermal conductivity increased with BN content in the usual way, and at the highest filler content of 34.2 vol% (approximately 49 wt.%) a value of 4.42 W/mK was measured, compared with a value of only 1.16 W/mK for a randomly dispersed sample. This represents an increase from somewhat above the lower trend line in Figure 9 for the random sample to just above the intermediate trend line for the 3-dimensionally structured sample (open dark brown square). In comparison with the values of thermal conductivity obtained for other epoxy-BN composites prepared by the aerogel procedure and discussed above, this result is not so noteworthy for such a high filler content. It is also worth remarking that, although the cure schedule only reached 160 °C as a maximum, these authors reported glass transition temperatures for the composite of up to 240 °C and decreasing markedly as the BN content increased; it is difficult to reconcile these observations.

**Figure 12:**
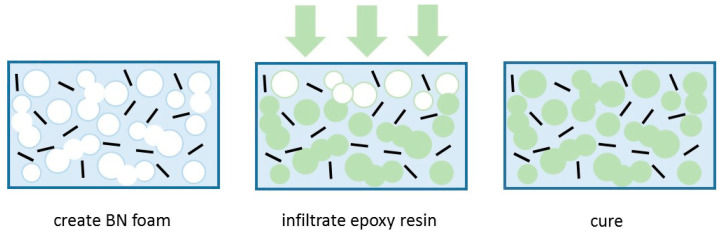
Schematic illustration of the fabrication of porous BN foam and epoxy-BN composite.

The above procedures usually introduce some anisotropy of thermal conductivity in the cured epoxy-BN composite. Tian et al. [132] argue that such anisotropy is not always desirable and describe an alternative way in which BN can be incorporated into a 3-dimensional structure, but with an isotropic thermal conductivity. These authors added BN particles to an aqueous solution of sodium dodecyl sulphate, which acts as both a foaming agent and surfactant, and gelatine, which provides the structural integrity of the BN foam. Foaming was induced by rapid stirring, and the slurry was then poured into a mould and stored at low temperature to solidify. After further drying at 70 °C, the previously mixed epoxy, anhydride curing agent and imidazole catalyst were poured into the porous BN foam, degassed, and then cured at 120 °C for 1 h and 160 °C for 3 h to obtain the epoxy-BN composite. The procedure is schematically illustrated in Figure 12. X-ray tomography shows that the BN filler formed a continuous 3-dimensional structure and that the epoxy filled the pores.

This procedure resulted in some remarkable thermal conductivity values. For composites fabricated with 24.4 wt.% of 8.7 μm BN platelets, the thermal conductivity in the in-plane direction was 5.19 W/mK and in the through-plane direction was 3.48 W/mK. In principle, this fabrication procedure should not give rise to anisotropic thermal conductivity; while not exactly isotropic, both of these values represent high thermal conductivities for the given filler content, as can be seen in Figure 9 (dark blue symbols), the former even lying well above the upper trend line in Figure 7, while the latter falls almost on it. Considerably lower values of thermal conductivity were found for composites fabricated in the same way but using smaller BN particles of sub-micron size (0.7 μm); at 25.1 wt.% filler content, these composites had a thermal conductivity of only 1.27 W/mK, just on the intermediate trend line in Figure 9 (open dark blue circle). The authors attributed the difference between the results for the micron-sized and sub-micron-sized BN particles to an alignment of the larger particles within the pore walls in the foam structure, in comparison with a random distribution for the sub-micron particles. It should be noted that these filler contents represent approximately limiting values for this procedure; according to the authors, any further increase of loading results in serious aggregation of the BN particles and poor structural uniformity of the composite. This can be noted from their graph of thermal conductivity as a function of BN content, for which there is a noticeable downward curvature at the highest contents.

A similar approach was adopted by Wang and Wu [135]. Melamine foam with an average pore size of ≈100 μm was repeatedly immersed in an aqueous solution of polyethyleneimine and then in a dispersion of exfoliated BNNS in isopropanol. In this way, they achieved a layer-by-layer deposition of BNNS on the melamine foam structure by virtue of the electrostatic attraction between the positively charged polyethyleneimine and the negatively charged BNNS, with up to 30 deposition cycles. Scanning electron microscopy and element mapping show that the BNNS coat the surfaces of the melamine foam cells rather homogeneously. The epoxy resin and curing agent, *o*-dichloroaniline methane, were then infiltrated into the foam under vacuum, followed by degassing and finally curing at 150 °C for 2 h and 180 °C for 2.5 h. It was found that the thermal conductivity increased with the number of deposition cycles, reaching a value of 0.53 W/mK after 20 cycles. Increasing the number of deposition cycles to 30 further increased the thermal conductivity to 0.6 W/mK, but the relative increase was markedly reduced, similar to the downward curvature in the results of Tian et al. [132]. Nevertheless, this value remains remarkable. The authors estimated the filler content to be about 1.1 vol%, approximately 2 wt.% calculated on the basis of the densities, and it can be seen that this value at a very low filler content lies well above the intermediate trend line in Figure 9 (open purple square). Indeed, the authors point out that to achieve this thermal conductivity with a random dispersion of BNNS in epoxy would require a filler loading of almost 20 vol%, which would correspond closely with the lower trend line in Figure 9.

### 4.3. Surface Decoration

There are a number of recent reports in which the surfaces of BN particles have been decorated with a variety of different nanoparticles or nanostructures, and following a variety of different procedures, some much more elaborate than others, with a view to achieving increased thermal conductivity. This has met with mixed success. Furthermore, since many of these decorating particles are themselves thermally conducting, such as graphene, silver and gold, the corresponding composites are no longer strictly epoxy-BN composites, but hybrids, and direct comparison with epoxy-BN composites fabricated in different ways is no longer valid. Nevertheless, some of these reports are considered here for the sake of interest.

Some of the simpler procedures were used by Han et al. [84] and Zhang et al. [155]. In the former case, hetero-structured SiC-BNNS fillers, fabricated by sol-gel and in situ growth methods, were mechanically mixed with the epoxy resin and curing agent and cured at 120 °C for 5 h. SEM images showed SiC nanoparticles on the surface of the BNNS, and that the number of SiC nanoparticles increased with the amount of Si powder used in the fabrication process. The mass proportions of SiC:BNNS used were 2:1, 1:1 and 1:2. The thermal conductivity of the epoxy alone was 0.22 W/mK, and those of the epoxy-SiC and epoxy-BNNS composites with 20 wt.% filler were 0.43 W/mK and 0.61 W/mK, respectively; this simply reflects the higher thermal conductivity of BN in comparison with SiC. There was a synergistic effect with the SiC-BNNS fillers, the thermal conductivity being higher than that of the composites with each filler separately. The optimum proportion was 1:1, at which a thermal conductivity of 0.89 W/mK was achieved at 20 wt.% filler content, but this value only just reaches the intermediate trend line in Figure 9.

Zhang et al. [155] used hybrids of small h-BN particles and larger cubic BN (c-BN) spherical agglomerates with a diameter of 59 μm. The procedure used was to modify the particles with a silane coupling agent, APTES, and then add them to an Au salt preloaded micellar solution to give Au-decorated BN. These particles were added in various proportions to the epoxy and curing agent in an ethanol solution, mixed thoroughly, degassed under vacuum, and then cured, first at room temperature for 3 h and then at 80 °C for a further 4 h. Similar to the findings of Han et al. [84], a synergistic effect was again observed: the thermal conductivity enhancement for 6 vol% was 50% for c-BN and 80% for h-BN, but a significantly higher enhancement of 120% was found for a hybrid with 4 vol% h-BN and 2 vol% c-BN. Despite this, the values of thermal conductivity reported are disappointing: 0.512 W/mK for a composite with 10.5 vol% (approximately 17 wt.%) filler without Au-decoration, increasing to 0.656 W/mK with Au-decoration, only just reaching the intermediate trend line.

Feng et al. [75] used a more elaborate fabrication procedure, devised to enhance both thermal conductivity and flame retardancy. By means of a simple hydrothermal process, they produced Ni(OH)_2_ nanoribbons that were 15 to 20 nm wide and 1 to 2 μm long, the purpose of which was to provide flame retardancy by endothermic decomposition. These nanoribbons were coated onto reduced graphene oxide (RGO) sheets by electrostatic self-assembly to give what the authors denoted as RGO@Ni(OH)_2_. An ethanol solution of RGO@Ni(OH)_2_ was mixed with an acetone solution of the diglycidyl ether of bisphenol-F epoxy and imidazole curing agent, the solvents were evaporated and then the 5 to 10 μm h-BN platelets were dispersed in the mixture and the composite was cured following a schedule of 2 h at 60 °C, 2 h at 100 °C, and 5 h at 150 °C. Composites were fabricated with up to 40 wt.% h-BN. Compared with composites of epoxy and h-BN platelets alone, for which a thermal conductivity of 1.44 W/mK was obtained for 40 wt.% filler content, composites with the same content of h-BN and only 2 wt.% RGO@Ni(OH)_2_ had a thermal conductivity of 2.00 W/mK, an increase of nearly 40%. In fact, a significant increase upon addition of 2 wt.% RGO@Ni(OH)_2_ occurred only with h-BN contents greater than 20 wt.%. The authors explained this on the basis of the dispersion of the h-BN platelets being too “dilute” at low filler contents, whereas at higher filler contents the RGO@Ni(OH)_2_ forms bridges between the h-BN platelets, creating thermal conduction pathways. Nevertheless, with respect to the thermal conductivity, even though this value is almost an order of magnitude greater than that of the epoxy alone (0.21 W/mK), at a filler content of 40 wt.% it still falls only about mid-way between the lower and intermediate trend lines.

Significantly more enhancement of thermal conductivity was achieved by Fu et al. [78], though at a much lower filler content of 4.7 vol%, through a combination of orientation and decoration procedures. BNNS were decorated with 10–20 nm Ag nanoparticles, denoted Ag-BNNS, and mixed with Ag nanowires (AgNW), previously synthesised by a modified polyol procedure, to give Ag-BNNS/AgNW. This mixture was ice-templated by freezing and freeze-drying to give a 3-dimensional network, which was then infiltrated by epoxy resin and the anhydride curing agent and cured for 2 h at 160 °C. The BNNS content was 4.7 vol%, which corresponds to approximately 8 wt.%. The thermal conductivity in the out-of-plane direction, which is the orientation direction of the BNNS, increased with AgNW content, which rose to 40 wt.% with respect to the BNNS content. The thermal conductivity at approximately 8 wt.% filler content was 0.34 W/mK for the epoxy-BNNS composite with untreated BNNS particles, 0.50 W/mK for epoxy/Ag-BNNS, and 0.80 W/mK for epoxy/Ag-BNNS/AgNW with the highest nanowire content. Although the thermal conductivity was low, as a consequence of the filler content being very low, these represent significant enhancements, the value of 0.80 W/mK falling well above the intermediate trend line, as can be seen in Figure 9 (filled green square). The authors attributed the enhancement to AgNW improving heat transfer between BNNS, termed a “physical connection”, and the Ag-decoration improving the interface between the BNNS and the AgNW, termed a “welding connection”. It should be borne in mind that these composites, even without the AgNW, are hybrids with the Ag nanoparticles decorating the surface of the BNNS, and direct comparison of their thermal conductivity with the other epoxy-BN composites discussed here should be made with caution.

The combination of surface decoration and a “densely-assembled and oriented” structure was introduced in an innovative way by Liu et al. [117], making use of 3-D printing technology, in order to optimise the thermal pathways and promote efficient heat transfer within the epoxy-BN composite. The 15 μm h-BN platelets were first exfoliated by sonication in dimethylformamide, and then aqueous AgNO_3_ was gradually added and stirred for 1 h at 150 °C to reduce the Ag^+^ to Ag nanoparticles (AGNPs) decorating the BN surfaces; this filler is denoted BN-Ag. The printing inks were prepared by mixing the DGEBA epoxy and amine curing agent with silica, as a thixotropic agent, to give a modified epoxy (MEP), and then adding the BN or BN-Ag particles to give loadings of 5, 10 and 20 wt.% in the final composites. The 3-D printing was carried out using single-material and multi-material printing. For single-material printing (S-BN/MEP and S-BN-Ag/MEP for undecorated and decorated BN platelets, respectively), the sample was printed by repeated passes of the MEP ink with the above filler loadings. For the multi-material printing (M-BN-Ag/MEP), the passes were made successively of MEP ink loaded with 40 wt.% BN-Ag filler and 1, 3 or 7 passes of MEP ink alone. This procedure gave the same 5, 10 and 20 wt.% filler loadings in the final composite, but with the conducting pathways “densely-assembled and oriented”. High resolution SEM and XRD show that the BN particles were oriented along the printing direction by shear alignment. The printed samples were cured for 3 h at 40 °C and 6 h at 80 °C. The thermal conductivity was measured in two orthogonal directions—the printing direction (PD) and the transverse direction (TD)—and in all cases was found to increase with BN content. In the TD, all the samples had approximately the same thermal conductivity, reaching 0.6 to 0.7 W/mK at a filler loading of 20 wt.% in comparison with 0.23 W/mK for the MEP alone. In the PD, on the other hand, there was a much more dramatic increase in the thermal conductivity with filler loading, increasing in the order S-BN/MEP (0.98 W/mK) → S-BN-Ag/MEP (1.77 W/mK) → M-BN-Ag/MEP (2.52 W/mK). The increase from S-BN/MEP to S-BN-Ag/MEP can be attributed to the presence of silver nanoparticles distributed on the surface of BN-Ag platelets, which reduce the contact thermal resistance, while the increase from S-BN-Ag/MEP to M-BN-Ag/MEP results from the dense filler loading of 40 wt.% in the heat-conducting phase, such that there is always contact between BN platelets. Once again, it should be borne in mind that these composites with BN-Ag are hybrids, with the Ag nanoparticles providing thermal pathways in addition to the BN. It is at least partly for this reason that the value of 2.52 W/mK for M-BN-Ag/MEP falls on the upper trend line in Figure 7, and the value of 1.77 for S-BN-Ag/MEP falls well above the intermediate trend line in Figure 9 (filled sage green symbols). On the other hand, without the Ag decoration on the BN platelets, the value of 0.98 W/mK for S-BN/MEP falls just on the intermediate trend line in Figure 9.

## 5. Concluding Remarks

This review has examined the cure kinetics and thermal conductivity of a large number of epoxy-BN composites reported in the literature. As regards the cure kinetics, the effect of fillers in general is most commonly to accelerate the cure reaction, usually attributed to the catalytic effect of, for example, hydroxyl groups present on the surfaces of the filler particles. For epoxy-BN composites in particular, there are rather limited studies of the cure kinetics, but it is interesting to note that for these epoxy-BN systems the reaction is more often retarded than accelerated by the filler particles. Since many of these cure kinetics studies of epoxy-BN systems involve thiol as a curing agent, it is surmised that this might be a consequence of a Lewis acid-base interaction between matrix and filler, and that this interaction might have some corresponding influence on the thermal conductivity of the cured composites. It transpires that such composites do indeed have a thermal conductivity which is higher than that of many other systems with the same filler content.

The discussion of the thermal conductivity considers composites which have been fabricated using a wide variety of methods, from simple mixing to much more elaborate procedures involving such aspects as orientation and other means of creating improved pathways for heat conduction. The composite systems include diverse epoxy resins and curing agents, and incorporate BN particles of different sizes and shapes, both with and without surface treatment and/or coupling agents. The dependence of the thermal conductivity on BN content in almost all cases follows the same tendency, namely to increase with increasing filler content and displaying an upward curvature. Within this broad tendency, however, there is great variation, and we attempt to analyse the reasons for this variation by defining three trend lines in the graph of thermal conductivity as a function of wt.% BN. The lower trend line indicates the approximate limit below which very few results lie; the vast majority of values of thermal conductivity reported for composites prepared without “special procedures” or orientation of the BN platelets lie between the lower and intermediate trend lines; and the upper trend line represents the approximate limit below which 95% of the reported values lie.

By locating reported values of thermal conductivity with respect to these trend lines it is possible to discuss the effects of the many variables involved. For example, the use of surface treatments and/or coupling agents nearly always increases the thermal conductivity. Thermal conductivity generally increases with BN particle size, and agglomerates are often better than platelets for any given filler content. To obtain thermal conductivities above the intermediate trend line usually requires some special preparation procedures, or the introduction of orientation of the BN platelets, though there are a few notable exceptions in which very high thermal conductivities were obtained without such procedures. Furthermore, in a majority of cases these special procedures apply to rather low filler contents, for example less than 20 wt.%, for which even a very significant increase in thermal conductivity relative to a simple mixing procedure results in a thermal conductivity which is still less than about 3 W/mK.

In general, if the use of these epoxy-BN composites is considered for application in IMS devices, there are some fundamental requirements that must be met, including high thermal conductivity and ease of processing. Many of the notable reports of enhancement of thermal conductivity discussed here, and principally those involving special procedures, do not meet either of these requirements, while many other procedures involve the use of solvents in the preparation of the composites, a practice which is not desirable for industrial applications. Likewise, from a consideration of the intermediate trend line, it is evident that to obtain a thermal conductivity greater than, for example, 5 W/mK using “simple” preparation techniques would require a filler content greater than about 50 wt.%, which also introduces practical difficulties as a consequence of the reduced manageability of such stiff pastes.

For IMS devices, therefore, there may be several procedures that could offer some practical advantages in attempting to achieve higher thermal conductivity. One is the use of pressure, which can be applied in practice quite easily, for example in an autoclave, and which allows the consolidation of relatively stiff resin-filler mixtures and elimination of voids. Another is to induce orientation in a type of pre-preg, which could then be laid up in a manner similar to that used in the fabrication of fibre-reinforced composites, to obtain the orientation in the required direction of heat transfer. Finally, the use of hybrids, for which several examples discussed exhibited synergistic behaviour, could provide a relatively simple means of enhancing the thermal conductivity, with combinations of different particle sizes and shapes. To date, it does not appear that these approaches have been exhausted.

## Supplementary Materials

The following are available online at https://www.mdpi.com/1996-1944/13/16/3634/s1, Supplementary Material 1: Excel spreadsheets giving correspondence between symbols and references in Figure 3 and Figure 4. Supplementary Material 2: Excel spreadsheets giving correspondence between symbols and references in Figure 7. Supplementary Material 3: Excel spreadsheets giving correspondence between symbols and references in Figure 9.
